# The origin of multicellularity in cyanobacteria

**DOI:** 10.1186/1471-2148-11-45

**Published:** 2011-02-14

**Authors:** Bettina E Schirrmeister, Alexandre Antonelli, Homayoun C Bagheri

**Affiliations:** 1 Institute of Evolutionary Biology and Environmental Studies, University of Zurich, Zurich, Switzerland; 2 Institute of Systematic Botany, University of Zurich, Zurich, Switzerland; 3 Current Address: Gothenburg Botanical Garden, Göteborg, Sweden

## Abstract

**Background:**

Cyanobacteria are one of the oldest and morphologically most diverse prokaryotic phyla on our planet. The early development of an oxygen-containing atmosphere approximately 2.45 - 2.22 billion years ago is attributed to the photosynthetic activity of cyanobacteria. Furthermore, they are one of the few prokaryotic phyla where multicellularity has evolved. Understanding when and how multicellularity evolved in these ancient organisms would provide fundamental information on the early history of life and further our knowledge of complex life forms.

**Results:**

We conducted and compared phylogenetic analyses of 16S rDNA sequences from a large sample of taxa representing the morphological and genetic diversity of cyanobacteria. We reconstructed ancestral character states on 10,000 phylogenetic trees. The results suggest that the majority of extant cyanobacteria descend from multicellular ancestors. Reversals to unicellularity occurred at least 5 times. Multicellularity was established again at least once within a single-celled clade. Comparison to the fossil record supports an early origin of multicellularity, possibly as early as the "Great Oxygenation Event" that occurred 2.45 - 2.22 billion years ago.

**Conclusions:**

The results indicate that a multicellular morphotype evolved early in the cyanobacterial lineage and was regained at least once after a previous loss. Most of the morphological diversity exhibited in cyanobacteria today —including the majority of single-celled species— arose from ancient multicellular lineages. Multicellularity could have conferred a considerable advantage for exploring new niches and hence facilitated the diversification of new lineages.

## Background

Cyanobacteria are oxygenic phototrophic prokaryotes from which chloroplasts, the light harvesting organelles in plants, evolved. Some are able to convert atmospheric nitrogen into a form usable for plants and animals. During Earth history, cyanobacteria have raised atmospheric oxygen levels starting approximately 2.45 - 2.22 billion years ago and provided the basis for the evolution of aerobic respiration [1-7]. Cyanobacteria have also evolved extensive morphological diversity. Various patterns of cell organization exist, ranging from single-celled to differentiated multicellular forms with branching patterns. Species of this phylum occupy various habitats. They can be found in marine, freshwater or terrestrial environments, ranging from polar to tropical climate zones. Based on their morphology, they have been divided into five sections [8,9] (Table 1). Sections I and II comprise single-celled bacteria, whereas sections III to V comprise multicellular forms. The latter sections are distinguished according to their level of organization. Section III is multicellular and undifferentiated, sections IV and V are multicellular and differentiated. The latter have the ability to produce heterocysts for nitrogen fixation and akinetes (climate-resistant resting cells). In addition, species in section V have the ability to branch in multiple dimensions.

**Table 1:** Subset of cyanobacterial taxa used for the analyses with GenBank accession numbers for 16S rDNA sequences

unicellular strains	accession numbers	multicellular strains	accession numbers
**Section I**		**Section III**	
*Chamaesiphon subglobosus *PCC 7430^1^	AY170472	*Arthronema gygaxiana *UTCC 393	AF218370
*Cyanobium sp*. JJ23-1	AM710371	*Arthrospira platensis *PCC 8005	X70769
*Cyanothece sp*. PCC 8801^1^	AF296873	*Crinalium magnum *SAG 34.87	AB115965
*Chroococcus sp*. JJCM	AM710384	*Filamentous thermophilic cyanobacterium*	DQ471441
*Dactylococcopsis sp*.^1^	AJ000711	*Geitlerinema sp*. BBD HS217^1^	EF110974
*Gloeobacter violaceus *PCC 7421^1^	BA000045	*Halospirulina sp*.^1^	NR_026510
*Gloeothece sp*. PCC 6909/1^1^	EU499305	*Leptolyngbya sp*. ANT.LH52.1	AY493584
*Microcystis aeruginosa *strain 038^1^	DQ363254	*Lyngbya aestuarii *PCC 7419^1^	AB075989
*Prochlorococcus sp*. MIT9313^1^	AF053399	*Microcoleus chthonoplastes *PCC 7420^1^	AM709630
*Prochloron sp*.^1^	X63141	*Oscillatoria sp*.^1^	AJ133106
*Radiocystis sp*. JJ30-3	AM710389	*Oscillatoria sancta *PCC 7515	AF132933
*Synechococcus elongatus *PCC 6301^1^	AP008231	*Phormidium mucicola *IAM M-221	AB003165
*Synechococcus sp*. CC9605	AY172802	*Plectonema sp*. F3^1^	AF091110
*Synechococcus sp*. WH8101	AF001480	*Planktothrix sp*. FP1	EU078515
*Synechocystis sp*. PCC 6803	NC_000911	*Prochlorothrix hollandica*^1^	AJ007907
*Synechocystis sp*. PCC 6308^1^	AB039001	*Pseudanabaena sp*. PCC 6802	AB039016
*Synechocystis sp*. CR_L29^1^	EF545641	*Pseudanabaena sp*. PCC 7304 ^1^	AF132933
*Synechococcus sp*. P1	AF132774	*Spirulina sp*. PCC 6313	X75045
*Synechococcus sp*. C9^1^	AF132773	*Starria zimbabweensis *SAG 74.90^1^	AB115962
*Synechococcus lividus *C1	AF132772	*Symploca sp*.PCC 8002	AB039021
*Acaryochloris sp*. JJ8A6^1^	AM710387	*Trichodesmium erythraeum *IMS 101^1^	AF013030
*Thermosynechococcus elongatus *BP-1^1^	BA000039	**Section IV**	
**Section II**		*Anabaena sp*. PCC 7108	AJ133162
*Chroococcidiopsis sp*. CC2	DQ914864	*Calothrix sp*. PCC 7103^1^	AM230700
*Dermocarpa sp*. MBIC10768	AB058287	*Nodularia sp*. PCC 7804^1^	AJ133181
*Dermocarpella incrassata*	AJ344559	*Nostoc sp*. PCC 7120	X59559
*Myxosarcina sp*. PCC 7312^1^	AJ344561	*Scytonema sp*. U-3-3^1^	AY069954
*Myxosarcina sp*. PCC 7325	AJ344562	**Section V**	
*Pleurocapsa sp*. CALU 1126	DQ293994	*Chlorogloeopsis sp*. PCC 7518^1^	X68780
*Pleurocapsa sp*. PCC 7516	X78681	*Fischerella sp*. PCC 7414	AB075986
		*Symphyonema sp*. strain 1517	AJ544084

		**Eubacteria**	
		*Beggiatoa sp. 'Chiprana'*	EF428583

^1^species used to test substitutional saturation

Different interpretations of multicellularity are currently used [10-12]. For cyanobacteria, characterization of multicellularity has been described in previous studies [13-16]. Cell to cell adhesion, intercellular communication, and for more complex species, terminal cell differentiation seem to be three essential processes that define multicellular, prokaryotic organisms on this planet [16]. Some forms of complexity found in several multicellular eukaryotes are not present in prokaryotes, but simple forms of multicellularity can be identified in three sections of the phylum cyanobacteria. Multicellular patterns comprise basic filamentous forms as found for section III, as well as more complex forms involving terminal differentiation, present in sections IV and V. In eukaryotes, multicellular complexity ranges from what is comparable to cyanobacteria to cases with up to 55 cell types as estimated for higher invertebrates such as arthropods or molluscs [17]. Considering that cyanobacterial sections III, IV and V resemble some of the first forms of multicellular filaments on Earth, knowing when and how these shapes evolved would further our understanding of complex life forms.

Some of the oldest body fossils unambiguously identified as cyanobacteria have been found in the Kasegalik and McLeary Formations of the Belcher Subgroup, Canada, and are evaluated to be between 1.8 billion and 2.5 billion years old [6,18]. Studies from ~ 2.0 billion year old formations [18,19] contain both unicellular and multicellular morphotypes of cyanobacteria. Cyanobacteria certainly existed as early as 2.32 billion years ago, if one accepts the assumption that they were responsible for the rapid accumulation of oxygen levels, known as the "Great Oxygenation Event" [1-3,5,7]. Multicellular fossils belonging to the cyanobacteria are well known from the late Precambrian [12,20,21] and possibly already existed 2.32 billion years ago. Other microbe-like multicellular filaments even older than 3.0 billion years have been found several times [22-26]. Some of the latter fossils are morphologically similar to species from the cyanobacterial order Oscillatoriales [27,28], but no clear evidence has been adduced yet. Although biogenicity of some of the oldest fossils has been questioned [29,30], a large variety of bacteria including anoxic phototrophs already existed by the time cyanobacteria evolved oxygenic photosynthesis [26]. Though impressive for prokaryotes, the fragmentary fossil record alone is not sufficient to disentangle the origin of cyanobacteria and their morphological phenotypes. Therefore, additional methods such as phylogenetic analysis provide a promising possibility to gather further clues on the evolution of such a complex phylum.

Phylogenetic analyses of cyanobacteria have gained in quantity over the past 20 years [4,31-39]. These studies have shown that morphological characterization does not necessarily reflect true relationships between taxa, and possibly none of the five traditional morphological sections is monophyletic. Similar morphologies must have evolved several times independently, but details on this morphological evolution are scarce. Analyses assessing characteristics of cyanobacterial ancestors [37,39] provide not only fundamental information on the history of cyanobacteria, but also on the evolution of life forms in the Archean Eon.

If one studies phylogenetic relationships based on protein coding genes in bacteria, it is possible to encounter the outcome of horizontal gene transfer (HGT) [40]. This issue is not as problematic for ribosomal DNA [41]. Nonetheless, the problem could be potentially reduced by analyzing datasets of concatenated conserved genes. Identification of these genes for phylogenetic analyses is not without difficulty, and requires in an ideal case comparison of complete genome data [42]. In cyanobacteria, many phylogenetic studies have concentrated on specific clades or smaller subsets of known species in this diverse phylum [39,43-48]. Therefore the genomic data presently available are strongly biased towards certain groups. In particular, genomic studies in cyanobacteria have emphasized marine species from Section I. Marine microphytoplankton (*Synechococcus *and *Prochlorococcus*) are a particularly well studied group [43,45,47,48], reflected by 19 sequenced genomes out of 41 cyanobacterial genomes sequenced to date (http://www.ncbi.nlm.nih.gov/genomes/lproks.cgi, accessed in January 2011). From species belonging to section III only two genomes (*Trichodesmium erythraeum *and *Arthrospira platensis*) are known. For sections IV (four genomes known) and V (no genomes known) molecular data are rare or missing. As genomic data accumulate, promising phylogenomic approaches to cyanobacteria are being established [37-39,47]. Despite these advances, it is at present difficult to obtain sequences other then 16S rDNA to cover a representative sample of species from all five sections.

The aim of this paper is to use molecular phylogenetic methods to address the evolutionary history of cyanobacteria and the evolution of multicellularity. For this purpose, we established a phylogeny based on 16S rDNA sequences belonging to 1,254 cyanobacterial taxa. From that phylogeny we sampled 58 cyanobacterial taxa that represent all main clades obtained and all five sections described by Castenholz *et al*. [8,9], and feature a 1:1 ratio of unicellular to multicellular species. We used several methods to reconstruct the morphological evolution of ancestral lineages, and compared our results to known fossil data. Since the fossil record is inconclusive on the timing and taxonomic position of multicellular cyanobacteria, our study provides independent evidence on the first appearance and evolution of multicellularity among the ancestors of living cyanobacteria.

## Results and Discussion

### Phylogenetic analysis

#### Phylogenetic analyses of all identified cyanobacteria

**Figure 1:**
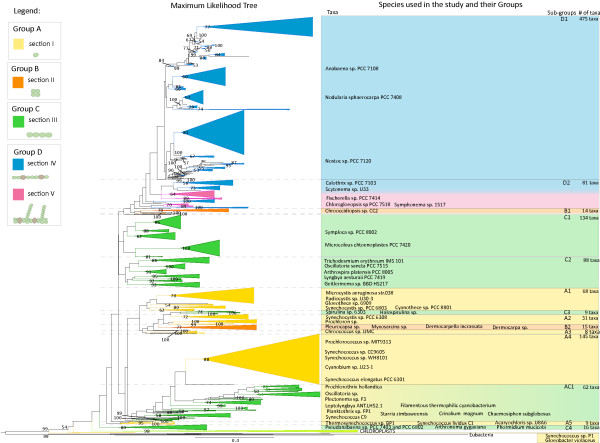
**Phylogenetic tree of 1,254 cyanobacterial species**. Maximum likelihood phylogram of cyanobacteria, based on GTR+G+I substitution model. Six eubacterial species form an outgroup. The ingroup contains 1,254 cyanobacterial strains and six different chloroplast sequences. Bootstrap values (> 50%) calculated from 100 re-samplings are displayed at the nodes. Colors define major morphological characters in the groups. Yellow are single-celled cyanobacteria of section I; orange single-celled from section II; green are multicellular, undifferentiated cyanobacteria from section III; blue are multicellular and differentiated bacteria from section IV; and pink from section V. Sections as described by Castenholz 2001 [9]. Different sub-groups (AC1;A1-A5;B1, B2;C1-C4;D1-D2) are defined for the phylogeny. Nomenclature of groups correlates with morphological sections as illustrated in the legend. From these sub-groups taxa were sampled for further analyses. A complete list with species included in the analysis can be found in Additional File 7.

To infer the evolution of multicellularity in cyanobacteria we carried out several phylogenetic analyses. To ensure a correct taxon-sampling, a phylogeny containing 1,254 16S rDNA sequences of cyanobacteria obtained from GenBank was reconstructed (Figure 1). Cyanobacterial morphotypes were assigned to four groups (A-D) which correlate to the five sections described by Castenholz *et al*. [9]. Using this nomenclature, sub-groups in the phylogeny were assigned to one of the four different morphological groups (A-D) according to their dominant shape. In total 14 sub-groups were identified for the phylogenetic tree. Five sub-groups consist of unicellular species from section I (A1-A5), two sub-groups are composed of single celled section II bacteria (B1, B2), four sub-groups are made up of multicellular species belonging to section III (C1-C4) and two sub-groups cover differentiated species from section IV and V (D1-D2). One sub-group contains both species from section I and III and is therefore designated as AC1. The phylogeny further contains six chloroplast genomes from the eukaryotic phyla Glaucophyta, Rhodophyta and the division Chlorophyta. Chloroplast sequences branch close to the bases and form a sister group to the cyanobacterial sub-groups mentioned. Furthermore six different Eubacteria were included in the phylogeny. They appear to form a distinct outgroup to the cyanobacteria and chloroplasts.

#### Phylogenetic analyses to identify an outgroup

**Figure 2:**
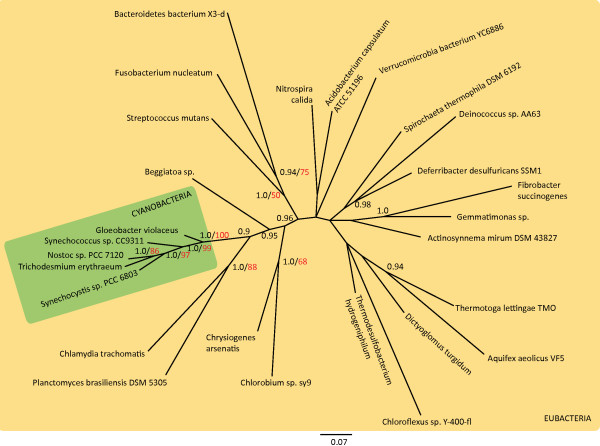
**Unrooted Bayesian consensus tree of Eubacteria including five cyanobacterial species**. Unrooted phylogenetic tree of 16S rRNA gene sequences from 27 eubacterial species reconstructed using Bayesian methods. Posterior probabilities (black) and bootstrap values (red) from 100 re-samplings are displayed at the nodes. Cyanobacteria, represented by 5 species, form a monophyletic group with *Gloeobacter violaceus *being closest to other eubacterial species.

Rooted and unrooted phylogenetic analyses reconstructed with maximum likelihood and Bayesian inference and based on 16S rRNA gene sequences of 27 eubacterial species, including 5 cyanobacteria revealed congruent results. Cyanobacteria form a monophyletic group. Figure 2 shows the unrooted Bayesian consensus tree which supports cyanobacterial monophyly with posterior probabilities (PP)/bootstrap values (BV) of 1.0/100%. Phylogenetic trees constructed with an archaean outgroup support cyanobacterial monophyly with PP/BV of 1.0/98% (Additional File 1). In both cases, *Plantomyces brasilienses *and *Chlamydia trachomatis*, both gram negative bacteria, form a sister group to the cyanobacteria. This does not agree with other studies [49-52], where *Deinococcus-thermus *was suggested to be the closest eubacterial relative to cyanobacteria. These discrepancies may be due to a lack of information when solely using 16S rRNA gene sequences for such distant relations. Furthermore, our results confirm the basal position of *Gloeobacter violaceus*, closest to the rest of the eubacteria, as found elsewhere [51]. This supports previous findings which state that *Gloeobacter violaceus *diverged very early from cyanobacteria living today [32,33,53,54]. *Gloeobacter *shows differences in cell structure and metabolism that clearly distinguish it from the rest of extant cyanobacteria [55,56]. It lacks thylacoid membranes and many genes from Photosystems I and II. Phylogenetic relations of the other eubacterial species show only weak support and are therefore not discussed further.

We separately tested each of the 22 eubacterial species originating from a diverse set of non-cyanobacterial phyla, with a subset of the cyanobacteria (58 taxa). The latter were chosen from the large dataset containing 1,254 taxa, and cover all sub-groups of the tree (Table 1). This subset was used for all subsequent phylogenetic analyses. Though multicellular species seem to dominate the known cyanobacteria, we chose to sample a taxa set containing unicellular and multicellular morphotypes in a 1:1 ratio. That way biases towards certain character states would be excluded. Furthermore, taxa used in the analyses should represent species from all five sections described by Castenholz *et al*. [9]. Given our interest in the base of the phylogeny, a greater number of taxa were sampled from basal sub-groups. Due to a lack of data available on GenBank at the present state of research, efforts to build a phylogenetic reconstruction of this size (58 species) using additional ribosomal protein sequences failed. But genomic data are accumulating (57 genomes in progress according to GenBank) and will soon offer possibilities for further extensive analyses.

**Figure 3:**
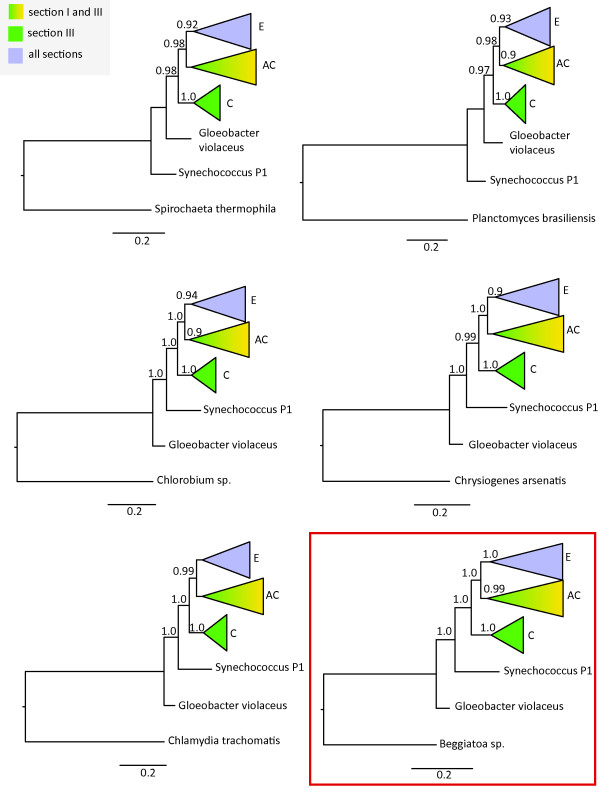
**Bayesian consensus trees of cyanobacterial subset using dierent eubacterial outgroups**. Six out of 22 phylogenetic trees reconstructed with Bayesian inference. For each tree an outgroup from a different eubacterial phylum was chosen. Posterior probabilities are displayed at the nodes. Green color represents multicellular cyanobacteria from section III, green-yellow gradient covers species from unicellular section I and multicellular section III, and purple depicts all five different morphological sections present in cyanobacteria. The majority of outgroups exhibits a similar tree topology. For further analyses *Beggiatoa sp*. was selected as an outgroup.

Results of six phylogenetic trees are displayed in Figure 3 (Additional file 2: Newick format of all trees). The majority of the trees exhibit a topology that agrees with Figure 2, with the position of *Gloeobacter violaceus *close to the outgroup. Strong differences are found in group support within the trees. In 14 of the 22 trees, three nodes could be identified which lead to three clades, named here E (**E**ntire five sections(A-D)), AC and C (nomenclature as described for the large tree; Figure 1). *Gloebacter violaceus *and *Synechococcus P1 *are found at the base of the cyanobacterial phylogeny in 16 trees, from which 7 trees exhibit *Gloeobacter violaceus *closest to the eubacterial outgroup.

In total 14 trees showed congruent topologies. From the 14 eubacteria which have been used as an outgroup in these trees, we chose *Beggiatoa sp*. as an outgroup for further analyses because its 16S rRNA gene sequence exhibits the shortest distance to the cyanobacteria.

#### Phylogenetic analyses of a cyanobacterial subset

**Figure 4:**
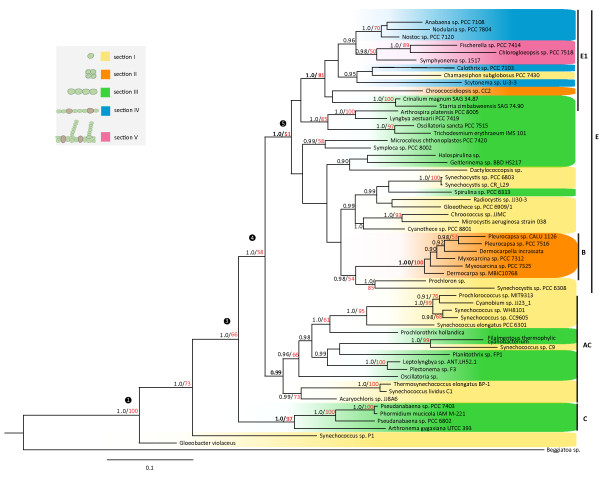
**Phylogenetic tree of a cyanobacterial subset**. Bayesian consensus cladogram of 16S rDNA sequences from 58 cyanobacterial strains, based on GTR+G+I substitution model, with *Beggiatoa sp*. used as outgroup. Posterior probabilities (> 0.9) are shown in black at nodes and bootstrap values (> 50%) in red. Posterior probabilities were calculated from 265,858 trees and bootstrap values from 500 re-samplings of the original data set. Colors define groups: yellow are single-celled cyanobacteria of section I; orange single-celled from section II; green are multicellular, undifferentiated cyanobacteria from section III; blue are multicellular and differentiated bacteria from section IV; and pink from section V. Sections as described by Castenholz 2001 [9]. AC, B, C, E and E1 denote phylogenetic clades described in the text.

Phylogenetic analyses of 16S rRNA gene sequences from a subset of 58 cyanobacterial taxa were conducted using maximum likelihood (Additional File 3) and Bayesian inference (Figure 4). For taxa that diverged a long time ago, there is a possibility of sequence saturation, in which case further mutations would have no effect on the distance between sequences any more. We could significantly reject the possibility of sequence saturation for our alignment (Additional File 4).

A general substitution model (GTR+G+I) was applied for both analyses. Results of the maximum likelihood and Bayesian methods are highly congruent. Result of the Bayesian analysis with posterior probabilities (black) and bootstrap values (red) displayed at the nodes is pictured in Figure 4. Posterior probabilities above 0.95 and bootstrap values over 70% are considered to represent a high phylogenetic support. Bootstrap values between 50% and 70% are considered weak support. Posterior probabilities below 0.90 and bootstrap values below 50% are not displayed. At deep nodes, the tree topology is fully resolved with high posterior probabilities. Apart from section V, none of the morphological sections described by Castenholz *et al*. [9] is monophyletic. Compared to the outgroup *Beggiatoa sp*., branch lengths are relatively short, which seems surprising given the old age of the phylum. Rates of evolution in cyanobacteria are extremely slow. This so called "hypobradytelic" tempo would explain their short evolutionary distances [20,57,58].

Cyanobacteria form three distinct clades mentioned earlier (Figure 3). Clades E, AC and C exhibit posterior probabilities (PP)/bootstrap values (BV) of 1.0/51%, 0.99/-, and 1.0/97% respectively (no support: "-"). Clade E comprises all taxa analyzed from section II, some from section I (*Synechocystis*, *Microcystis, Gloeothece *and others), some from section III (*Oscillatoria, Trichodesmium, Arthrospira, Lyngbya, Microcoleus, Spirulina *and others) and all from sections IV and V. Within clade E two subclades, E1 (species from section II; PP/BV = 1.0/81%) and B (species from sections IV and V among others; PP/BV = 1.0/100%), are found. Clade AC contains species from section I and III (among others, species from the genera *Synechococcus, Prochlorococcus, Oscillatoria, Plectonema*). Clade C consists of *Pseudanabaena *species, *Arthronema gygaxiana *and *Phormidium mucicola *belonging to section III. *Gloeobacter violaceus *is placed closest to the outgroup. Several phylogenetic studies were conducted showing approximate agreement with the tree topology generated here [4,31-39,54]. To check the consistency of results from the maximum likelihood and Bayesian analysis to previous studies, we compare our results to the trees produced by Honda *et al*. [32], Turner *et al*. [33] who used 16S rDNA sequences, and Swingley *et al*. [38] who used a genomic approach.

The tree from Figure 2 in Honda *et al*. [32] shows overall strong congruences with our tree. The only exception is that in Honda *et al*. [32] "*Synechococcus elongatus *Toray" is placed separately between *Gloeobacter *and the rest of the cyanobacteria. We found that "*Synechococcus elongatus *Toray" (identical to *Thermosynechococcus elongatus *BP1) is located within clade AC in our study and not next to *Gloeobacter violaceus*.

In Turner *et al*. [33], the major clades are congruent with those inferred in our study, but there are a few differences in the relationships among these clades. In that study, the analog of clade E1 is sister to clade AC, which is not the case in our consensus tree. Furthermore, *Synechococcus *C9 is grouped with *Synechococcus *P1, which might be due to long branch attraction. In our phylogenetic tree, *Synechococcus *C9 is grouped within clade AC, a relationship supported by high posterior probabilities and bootstrap values (1.0/99%). Clade C in our study is placed in the same position as in the tree from Turner *et al*. [33].

Swingley *et al*. [38], used a phylogenomic approach to investigate cyanobacterial relationships. Due to limited, biased genome data available at present, some clades present in our tree are missing in that study. Even so, the main clades retrieved in that study are mostly congruent with clades in our tree.

Monophyly of section V (the branching, differentiated cyanobacteria) shown in our tree agrees with Turner *et al*. [33] and other studies [36,54]. Nonetheless it is possible that the monophyly of section V bacteria is due to limited taxon sampling, since polyphyly has been detected for section V in another study [59]. *Gloeobacter violaceus *is placed as the first diverging lineage in the phylogeny after the outgroup, as suggested by previous studies [4,32-35,37,39,54]. Our phylogenetic reconstruction also confirms the placement of taxa belonging to section I and III throughout the tree [4,31-37,39,54]. The finding that possibly none of the traditional morphological sections are monophyletic, clearly indicates that similar morphologies have been gained and lost several times during the evolutionary history of living cyanobacteria. Overall, the strong phylogenetic agreement between this and earlier studies confirms the suitability of the tree presented here for further analyses of morphological evolution.

### Ancestral character state reconstruction

Our analysis indicates that multicellularity is a phylogenetically conservative character (p-value < 0.01). If the terminal taxa of the Bayesian consensus tree are randomly re-shuffled, a count through 1,000 re-shuffled trees gives an average of 20 transition steps. However an average of only nine parsimonious transitions was observed in a count through 10,000 randomly sampled trees of our ancestral character state reconstruction.

**Figure 5:**
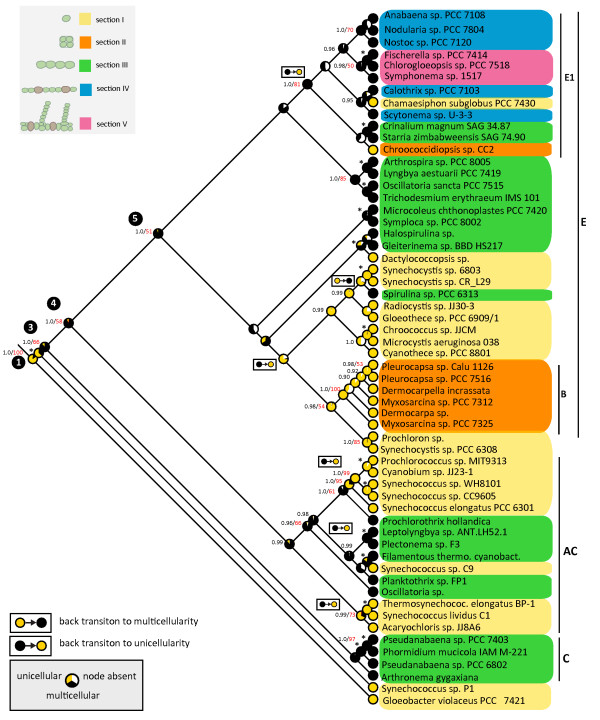
**Ancestral character state reconstruction using maximum likelihood**. Ancestral character state reconstruction with maximum likelihood analysis, using the "Asymmetrical Markov k-state 2 parameter"(AsymmMk) model implemented in Mesquite 2.71 [60]. Transition rates were estimated by the program (Table 2). Analysis was run over 10,000 randomly sampled trees from the Bayesian analysis and plotted on the Bayesian consensus tree. Possible states are unicellular (yellow) and multicellular (black). Relative likelihood probabilities for each character state are represented with a pie chart at nodes. The white part in the pie charts indicates the fraction of trees where the node was absent. Posterior probabilities (black) and bootstrap values (red) from the phylogenetic analyses are displayed at the nodes. Asterisks denote supported nodes for which posterior probabilities and bootstrap values are presented in Figure 4. At nodes 3, 4 and 5 a multicellular ancestry is very likely. Back mutations to unicellularity occur at least five times. A back mutation to multicellularity occurs at least once. Clades where transitions occurred are labelled.

Results of the character state reconstruction using the AsymmMK model with transition rates estimated by Mesquite 2.71 [60] are displayed in Figure 5. Using maximum likelihood analysis, average frequencies of the characters were counted across 10,000 trees randomly sampled from the two Metropolis-coupled Markov Chain Monte Carlo (*MC*^3^) searches of the Bayesian tree reconstruction.

Cyanobacteria share a unicellular ancestor, but multicellularity evolved early in the cyanobacterial lineage. We identified multicellular character states for three basic ancestors leading to clades E, AC and C in our tree. Together, these clades encompass the entirety of the morphological sections II, III, IV and V. Additionally character states were reconstructed using maximum likelihood analysis and fixed transition rates to analyze properties of the data set. Transition rates are presented in Table 2. Probabilities for character states at nodes 3, 4 and 5 were examined in detail (Table 3). A multicellular ancestry is very likely for these three nodes. For node 3 the relative probabilities of a multicellular ancestor range from 0.79 to 1.00, depending on the probability of the transition rates. For node 4 with varying transition rates, the relative probabilities of a multicellular ancestor range from 0.83 to 1.00. For node 5 the probabilities for multicellularity range from 0.90 to 1.00.

**Table 2:** Different Transition rates with whom ancestral character states were estimated.

method rates	Maximum likelihood analysis								Bayesian analysis
		
	AsymmMK^1^	MK1^2^	F1^3^	F2	F3	F4	F5	F6	rjhp^4^
**fw**^5^	1.62	2.67	0.90	2.70	5.40	0.45	0.90	2.70	2.881
**bw**^6^	2.99	2.67	2.70	0.90	0.45	5.40	0.90	2.70	2.873

^1^Asymmetrical Markov k-state 2 parameter model; rates estimated from the consensus tree

^2^Markov k-state 1 parameter model; rates estimated from the consensus tree

^3^F1-F6: Models using different fixed transition rates

^4^reversible jump for model selection, using a hyper prior

^5^forward rate describing changes to multicellularity

^6^backward rate describing changes back to a unicellular state

**Table 3:** Ancestral character states of nodes 3, 4 and 5 using different transition rates and methods.

			node 3		node 4		node 5	
					
method	model		state1	state0	state1	state0	state1	state0
**ML**^1^	AsymmMK	estimated^3^	0.88	0.12	0.91	0.08	0.95	0.05
		F1	0.96	0.04	0.98	0.02	0.99	0.01
		F2	0.87	0.12	0.91	0.09	0.94	0.06
		F3	1.00	0.00	1.00	0.00	1.00	0.00
		F4	0.88	0.12	0.92	0.08	0.95	0.05
	
	MK1	estimated^3^	0.79	0.21	0.83	0.17	0.90	0.10
		F5	0.88	0.12	0.90	0.10	0.93	0.07
		F6	0.79	0.21	0.83	0.17	0.90	0.10

**MP**^2^			0.6805	0.0013	0.6799	0.0014	0.6871	0.0014

**BA**^3^	rjhp		0.915	0.0851	0.817	0.183	0.902	0.0980

^1^Maximum likelihood: Average frequencies across trees were calculated

^2^Maximum parsimony: Uniquely best states across trees were counted

^3^Bayesian analysis: model parameters estimated based on the data

The maximum likelihood analysis is not contradicted by a Maximum Parsimony optimization (Table 3 and Additional File 5). Applying maximum parsimony as a reconstruction method, the uniquely best states were counted across 10,000 trees randomly sampled from the two (*MC*^3^) runs of the Bayesian tree reconstruction. The relative probabilities for a multicellular ancestor at nodes 3, 4 and 5 are 0.68, 0.68 and 0.69, respectively. In contrast, the relative probabilities for a unicellular ancestor at nodes 3, 4 and 5 under parsimony reconstruction are 0.0013, 0.0014 and 0.0014, respectively.

**Figure 6:**
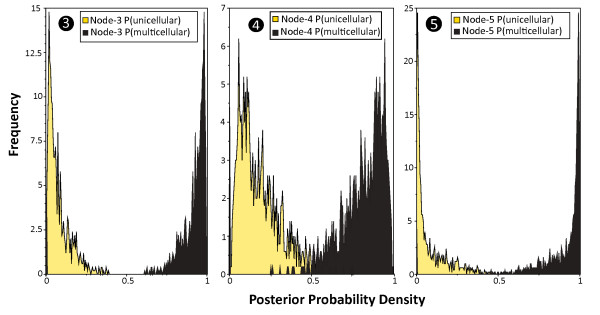
**Ancestral character states of nodes 3, 4 and 5 using Bayesian analysis**. Posterior probability distribution for a unicellular character state (yellow) and a multicellular character state (black) at nodes 3, 4 and 5 from 10,000 Bayesian trees. 2× 5,000 trees were randomly sampled from 2 *MC*^3^-searches. Analysis was performed using BayesTraits. Posterior distributions were derived from reversible jump MCMC-search of 30 million iterations using a hyperprior approach. The probability of a multicellular ancestry is shifted towards 1 for each of the three nodes.

Using Bayesian methods, a similar pattern is observed for these nodes. As an evolutionary model, BayesFactors revealed that a "hyperprior" approach with exponential prior distributions, whose means were sampled from a uniform distribution between 0 and 10 gave the best fit. Transition rates were estimated to be almost equal. Figure 6 displays the posterior probability distributions of character states at these three nodes as they were estimated over 10,000 randomly sampled trees. At nodes 3 and 5 posterior probabilities of a multicellular character state display values above 0.90 for most of the trees. At node 4 a multicellular state is more likely as well. Posterior probabilities at node 4 are above 0.75 for most of the trees.

At least five reversals to unicellularity occurred in the tree, three of them within clade AC. The first transition occurred on a branch which led to a group of thermophilic cyanobacteria: *Acharyochloris sp*., *Synechococcus lividus *C1 and *Thermosynechococcus elongatus*. Posterior probabilities (PP) and bootstrap values (BV) for this group are 0.99/73%, whereas the sister group within AC is supported by 0.96/66% (PP/BV). The second transition within clade AC led also to a thermophilic cyanobacterium *Synechococcus *C9. Sister relation of this species to a filamentous thermophilic cyanobacterium is supported by 1.0/99% (PP/BV). The last transition in clade AC occurred within the group including the marine pico-phytoplankton genera *Synechococcus *and *Prochlorococcus*. The filamentous *Prochlorothrix hollandica *is supposed to be the closest relative to the group that includes marine pico-phytoplankton, supported by 1.0/61% (PP/BV). Clade AC has a PP of 0.99, while its BV is below 50%. Although bootstrap support is below 70% for clade AC and some groups within it, posterior probabilities show a very high support (> 0.95). Simulation studies have shown that posterior probabilities approach the actual probability of a clade [61-63]. Bootstrapping tends to underestimate the actual probability of a true clade. Although, posterior probabilities tend to be erroneous if the model of evolution is underparameterized, overparameterization has only a minor effect on the posterior probabilities. Therefore, using a complex model of evolution, such as the "general time reversible with *gamma *distributed rate variation"(GTR+G), is recommended [62,63]. We used the GTR+G+I model for our analysis, and assume that nodes with a PP higher than 0.95 are reliable.

It is very likely that at least one additional reversal to unicellularity occurred in clade E1, but phylogenetic support is not high enough to locate the exact position of this transition. Similarly, support for the nodes where the other transition to multicellularity within clade E occurred is missing. The exact locations of reversals within clade E therefore are not certain and a scenario where multiple reversals occurred cannot be excluded. In clade E, there is also a reversal to multicellularity observed in *Spirulina sp*. PCC 6313. The location of this transition is supported by posterior probabilities of 0.99 at two ancestral nodes.

Stucken *et al*. [64] compared gene sets of multicellular cyanobacteria and found that at least 10 genes are essential for the formation of filaments. Besides genes previously thought to be correlated with heterocyst formation (*hetR*, *patU3 *and *hetZ*) they found seven genes coding for hypothetical proteins. The species they compare are all located within clade E in our tree, most of them being differentiated. Unfortunately no genomes from multicellular species in more basal clades are available at present. But genome projects of *Phormidium sp*. ISC 31 and *Plectonema sp*. ISC 33 are presently being conducted http://www.ncbi.nlm.nih.gov/genomes/lproks.cgi. If these species turn out to group with *Phormidium mucicola *IAM M-221 and *Plectonema sp*. F3 from the basal clades C and AC in our study, this could provide important information on the original metabolic pathways in ancient multicellular cyanobacteria and on possible advantages of multicellularity.

The majority of cyanobacteria living today are described as successful ecological generalists growing under diverse conditions [20]. Our analysis indicates that this diverse range of cyanobacterial morphotypes found in various habitats today —whether multicellular or unicellular— has evolved from multicellular ancestors.

### Gaining and losing multicellularity

In eukaryotes, simple multicellular forms build the foundation for the evolution of complex multicellular organisms. Although complex multicellularity exhibiting more than three cell types is presumably missing in prokaryotes, bacteria invented simple multicellular forms possibly more than 1.5 billion years earlier than eukaryotes [24-26,65]. Multicellularity has been described as one of several major transitions that occurred in the history of life. These transitions between different units of selection [66] resulted in changes in the organizational confines of the individual. Maynard Smith and Szathmary [67](1995, p.6) summarize eight major transitions in the evolution of life after which, "entities that were capable of independent replication before the transition can replicate only as part of a larger whole after it". These transitions can create new units of selection at a higher level of complexity [68]. Origin of chromosomes, origin of the eukaryotic cell, origin of multicellular organisms and the origin of eusocial communities are some major transitions that redefine the degree of individuality [66,67,69,70]. Some transitions are thought to be unique, such as the evolution of meiosis or the evolution of the genetic code. Other major transitions occurred several times independently, such as the evolution of eusociality [71,72] and multicellularity [10,66,73-75]. There is a tendency to assume that these transitions occur in a progression that leads to an increase in complexity. However, it seems that in cyanobacteria this is not the case. Anatomical complexity has been lost during their evolution several times (Figure 5). In a similar fashion, a complex character such as eusociality has been lost several times in halictid bees [72,76]. Conversely the phylogeny indicates that multicellularity re-evolved in *Spirulina*. Regaining complex characters has been observed in other studies as well [77-79]. Nonetheless, some studies state that re-evolution of a complex character after a previous loss is not possible [80,81]. Such studies argue that according to 'Dollo's law', a loss of complexity is irreversible [82], a statement that is not supported in the cyanobacterial case. Repeated transitions in either direction are possible.

### Prokaryotic fossil record before the "Great Oxygenation Event": Evidence for multicellular cyanobacteria?

**Figure 7:**
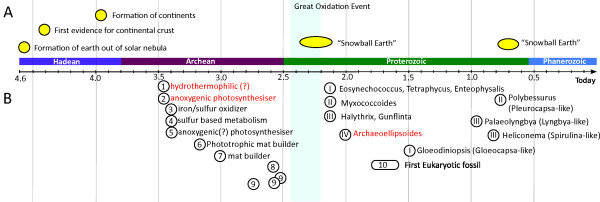
**Timeline with prokaryotic fossil record**. Timeline with geological events (A) and prokaryotic fossil record (B). (A) Formation of Earth [118], first evidence of continental crust [119], formation of continents [118], and glaciation events described in the Snowball Earth hypothesis [120]. (B) The oldest conclusive cyanobacterial fossils are found in around 2.15 billion year old rocks. 1-7: Fossils from the Archean Eon [23,25,26,84-87]. 8: chroococcacean fossils [24]; 9: oscillatorian fossils [24]. I-V: cyanobacterial fossils [18-20]. 10: eukaryotic fossils [65].

Various claims for life during the early Archean Eon, more than 3.00 billion years ago exist. Most of them from two regions: the Berberton Greenstone Belt, South Africa (around 3.20-3.50 billion years old) and the Pilbara Craton, Western Australia (around 2.90-3.60 billion years old). For some of these "fossils" a biological origin is questioned [26,27,83], but for others biogenicity is very likely [23,25,26,84-87]. These candidates for early life have clear age constraints and there is no non-biological explanation for these structures. The ages and possible metabolic features of seven fossils of proposed biological origin are plotted in Figure 7 (1-7) [23,25,26,84-87]. Some of these fossils are assumed to have been photosynthetic and mat builders, characteristics that can be identified in cyanobacteria as well. One of the oldest fossils recorded, 3.45 billion year old prokaryotic remains found in the Panorama Formation, East Pilbara Craton, Western Australia exhibit a filamentous morphotype and possibly carried out anoxygenic photosynthesis [25,26].

Some late Archean fossils show an oscillatorian or chroococcacean morphotype (Figure 7: 8, 9). 2.52 and 2.56 billion year old oscillatorian-like fossils [24,88,89] could possibly represent close relatives of cyanobacterial ancestors. 2.72 billion year old filamentous bacteria [24] could potentially represent one of the first multicellular cyanobacteria detected. For single celled forms, 2.56 billion year old unicellular fossils [89-92] could likely represent chroococcacean fossils, relatives of ancestral *Gloeobacter violaceus *or *Synechococcus sp*. P1 (Figure 7).

The first conclusive cyanobacterial fossils from all five sections have been reported from around 2.15 billion year old rocks. In 1976, Hofmann described Microfossils from stromatolitic dolomite stones in the Kasegalik and McLeary Formations of the Belcher Supergroup in Hudson Bay, Northern Canada. Among these fossils are *Halythrix *which seems to belong to the order Oscillatoriales (section III), *Eosynechococcus *and *Entophysalis *both presumably order Chroococcales (section I) and *Myxococcoides *fossils (section II). In 1997 similar fossils were described by Amard and Bertrand-Safarti in paleoproterozoic cherty stromatolites from the "Formation C (FC)" of the Franceville Group in Gabon, dating back 2.00 billion years. They also characterized chroococcalean fossils, particularly *Eosynechococcus *and *Tetraphycus*, and filamentous bacteria (*Gunflinta*) which could likely resemble cyanobacteria and *Myxococcoides *fossils. Furthermore, large microfossils (so called *Archaeoellipsoides elongatus*), with akinetes similar to the ones from Anabaena-like species were found [4,19]. Akinetes are resting cells which are only present in differentiated cyanobacteria from sections IV and V. As it has been confirmed in several studies, sections IV and V share a most recent common ancestor [4,33,36]. Therefore these fossil akinetes document the existence of differentiated cyanobacteria 2.00 billion years ago. Given that differentiation in cyanobacteria is evolutionary stable only in a multicellular setting [93], this again supports the notion that multicellular species belonging to the cyanobacteria must have existed earlier than 2.0 billion years ago.

Several studies have assessed prokaryotic history using phylogenetic dating methods [50,52]. In these studies the origin of cyanobacteria has been estimated around the time of the "Great Oxygenation Event" of 2.20-2.45 billion years ago [2,7]. Other studies have reported elevations of oxygen levels before the great rise of atmospheric oxygen [7,94]. Using small and large ribosomal subunit sequences, Blank and Sanchez-Baracaldo [39] estimated the origin of cyanobacteria between 2.7 and 3.1 billion years ago. They also try to address the evolution of cyanobacterial traits and assess that multicellular cyanobacteria did not originate before 2.29-2.49 billion years ago. In the study of Blank and Sanchez-Baracaldo [39], a smaller set of cyanobacterial taxa was used, with some basal multicellular species that are present in clade C of our analysis missing. These taxa could have an essential effect on the timing of the first multicellular cyanobacteria. To resolve this issue further dating analyses would be needed. Clearly, as Blank and Sanchez-Baracaldo point out, for such analyses to ultimately resolve the cyanobacterial history, a larger number of cyanobacterial genome data would be needed to represent all the morphological and genetic diversity within this phylum.

## Conclusions

Cyanobacteria, photosynthetic prokaryotes, are one of the oldest phyla still alive on this planet. Approximately 2.20-2.45 billion years ago cyanobacteria raised the atmospheric oxygen level and established the basis for the evolution of aerobic respiration [1-6]. They introduced a dramatic change in the Earth's atmosphere, which might have created possibilities for more complex lifeforms to evolve. Considering the importance of cyanobacteria for the evolution of life, it seems unfortunate that data sets for a representative phylogenomic analysis are not yet available. A coordinated perspective between research groups and a diversified taxon sampling strategy for genome projects would offer the possibility for more comprehensive studies on cyanobacterial evolution. By presenting results obtained from 16S rDNA data analysis here, we hope to boost interest for more extensive genomic studies in this phylum. Phylogenomic approaches would help to further investigate some of the results in the present work.

Multicellular prokaryotic fossils from the Archean Eon are documented [25,26], and fossil data can support the possibility of multicellular cyanobacteria in the Archean Eon [24,88-90]. Furthermore, studies describe smaller accumulations of oxygen levels around 2.8 to 2.6 billion years ago [7] and around 2.5 billion years ago [94]. Therefore multicellular cyanobacteria could have evolved before the rise of oxygen in the atmosphere. The "Great Oxygenation Event", also referred to as "oxygen crisis", could presumably have marked one of the first mass extinction events during Earth's history. New habitats developing around 2.32 billion years ago, due to a dramatic change of Earth's atmosphere could have triggered cyanobacteria to evolve the variety of morphotypes preserved until today.

In terms of cell types, cyanobacteria reached their maximum morphological complexity around 2.00 billion years ago [95]. By the time eukaryotes evolved, cyanobacteria already exhibited the full range of their morphological diversity. Due to slow evolutionary rates in cyanobacteria, which have been described as "hypobradytelic" [20,57,58], extant cyanobacteria that appear to exhibit the same morphotype as in the Precambrian Eon [96] are reminiscent of the idea of "living fossils". However, one should consider the possibility that what may appear as morphological stasis may be due to developmental constraints at the phylum level. Cyanobacteria apparently reached their maximum complexity early in Earth history, but instead of morphological stasis at the species level, our results suggest that they subsequently changed morphotypes several times during their evolution. This allowed for the exploration of diverse morphotypes within their developmental constraints, including the loss and regaining of multicellular growth forms.

**Figure 8:**
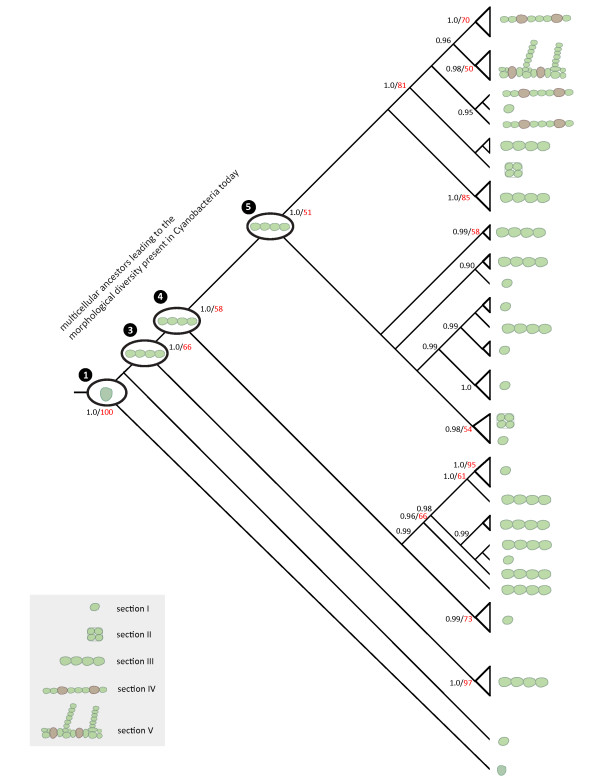
**Schematic illustration of cyanobacterial evolution**. Numbers at the nodes indicate Bayesian posterior probabilities (black) and bootstrap values (red) from the phylogenetic analyses. The most recent common ancestor of all cyanobacteria is optimized to have been unicellular. All cyanobacteria derive from a unicellular most recent common ancestor (node 1). The lineage leading to *Gloeobacter violaceus *diverges very early from the remaining cyanobacteria. Most major clades of cyanobacteria derive from multicellular ancestors (nodes 3-5).

Figure 8 summarizes the morphological evolution of the cyanobacteria inferred in this study. All extant cyanobacteria share a most recent common ancestor that was unicellular. Single-celled species at the base of the tree do not seem to have changed much in their morphology and are possibly comparable to ancient cyanobacteria. Aside from *Gloeobacter violaceus *and *Synechococcus *P1, which diverged very early, all cyanobacteria living today share multicellular ancestors. Although complex multicellularity is missing in prokaryotes, these simple multicellular forms have evolved several hundred million years before the appearance of eukaryotes, whose fossil record dates back to 1.8-1.3 billion years ago [65]. In agreement with various proposed selective advantages that multicellular growth could confer [97-100], the results presented here indicate that the early origin of multicellularity played a key role in the evolutionary radiation that has led to the majority of extant cyanobacteria on the planet.

## Methods

### Taxon sampling

A total of 2,065 16S rRNA gene sequences from the phylum cyanobacteria were downloaded from GenBank. Unidentified and uncultured species were excluded. With this large dataset phylogenetic reconstructions were conducted as described in the next section. Aside from cyanobacteria, the dataset included six chloroplast sequences and six eubacterial sequences: *Beggiatoa sp*., *Thiobacillus prosperus*, *Agrobacterium tumefaciens*, *Chlorobium sp*., *Candidatus Chlorothrix halophila *and *Escherichia coli HS*.

From this large tree a subset of 58 cyanobacterial sequences were selected for further analyses. Accession numbers are provided in Table 1. Species from all five sections described by Castenholz *et al*. [9] were included. Taxa were chosen to represent a 1:1 ratio of unicellular and multicellular species. The final data set contained 22 single-celled taxa from section I, 7 single-celled taxa from section II, 21 multicellular taxa from section III, 5 multicellular, differentiated taxa from section IV and 3 differentiated, branching taxa from section V as described by Castenholz *et al*. [9].

An outgroup for further analyses was chosen from a set of eubacterial, non-cyanobacterial species whose 16S rRNA gene sequences were downloaded from GenBank (Table 4). Species were sampled to cover a wide range of different phyla. Aside a set of species from phyla represented in the "tree of life" [51], species from additional phyla as described on NCBI http://www.ncbi.nlm.nih.gov/Taxonomy/ - Taxonomy Browser: Bacteria) were selected for analyses.

**Table 4:** Non-cyanobacterial species used in this study with GenBank accession numbers for 16S rDNA sequences

	Phyla^1^	species	accession numbers
**EUBACTERIA**	Acidobacteria	*Acidobacterium capsulatum *ATCC 51196	CP001472
	Actinobacteria	*Actinosynnema mirum *DSM 43827	CP001630
	Aquificae	*Aquifex aeolicus *VF5	NC_000918
	Bacteroidetes	*Bacteroidetes bacterium *X3-d	HM212417
	Chlamydiae/Verrucomicrobia	*Chlamydia trachomatis*	AM884176
	Chlamydiae/Verrucomicrobia	*Verrucomicrobia bacterium *YC6886	FJ032193
	Chlorobi	*Chlorobium sp*. sy9	EU770420
	Chloroflexi	*Chloroflexus sp*. Y-400-fl	NC_012032
	Chrysiogenetes	*Chrysiogenes arsenatis*	NR_029283
	Deferribacteres	*Deferribacter desulfuricans *SSM1	AP011529
	Deinococcus-Thermus	*Deinococcus sp*. AA63	AJ585986
	Dictyoglomi	*Dictyoglomus turgidum*	NC_011661
	Fibrobacteres	*Fibrobacter succinogenes*	NC_013410
	Firmicutes	*Streptococcus mutans *NN2025	AP010655
	Fusobacteria	*Fusobacterium nucleatum*	GU561358
	Gemmatimonadetes	*Gemmatimonas sp*.	GU557153
	Nitrospirae	*Nitrospira calida*	HM485589
	Planctomycetes	*Planctomyces brasiliensis *DSM 5305	NZ_AEIC01000055
	Proteobacteria	*Beggiatoa sp*. 'Chiprana'	EF428583
	Spirochaetes	*Spirochaeta thermophila *DSM 6192	NC_014484
	Thermodesulfobacteria	*Thermodesulfobacterium hydrogeniphilum*	AF332514
	Thermotogae	*Thermotoga lettingae *TMO	NC_009828

**ARCHAEA**	Nanoarchaeota	*Nanoarchaeum equitans *Kin4-M,	NC_005213

^1^taxonomy as described at http://www.ncbi.nlm.nih.gov/Taxonomy/ and [51]

### Phylogenetic analyses

#### Phylogenetic analyses of all identified cyanobacteria

The 2,065 16S rRNA gene sequences were aligned using the software MAFFT [101] via Cipres Portal [102]. The alignment was corrected manually using BioEdit v7.0.5 [103]. Poorly aligned and duplicated sequences were excluded from the alignment. From the remaining 1,254 sequences (1235 characters) a phylogenetic tree was reconstructed running 10 maximum likelihood analyses as implemented in RAxML v7.0.4 [104]. GTR + G + I (General time reversible model, G: Gamma correction, I: proportion of invariable sites) [105,106] was used as an evolutionary substitution model. Bootstrap values were calculated from 100 re-samplings of the dataset and plotted on the best maximum likelihood tree using RAxML v7.0.4. The resultant tree (Figure 1; Additional File 6: newick format; Additional File 7: taxon names) was visualised in FigTree v1.3.1 http://tree.bio.ed.ac.uk/software/figtree/ and graphically edited with Adobe Illustrator CS2 http://www.adobe.com/products/illustrator/.

#### Phylogenetic analyses to identify an outgroup

To test different outgroups, phylogenetic trees were reconstructed using all sampled non-cyanobacterial species (Table 4) plus five representative species from the cyanobacterial phylum (Table 1). Sequences were aligned using Clustal-X with default settings [107] and corrected manually. The trees were built using maximum likelihood and Bayesian inference, with and without an outgroup from the kingdom archaea. Fifty separate maximum likelihood searches were conducted using RAxML v7.0.4 software [104], from which the tree with the best log-likelihood was chosen. Bootstrap support for each tree was gathered from 100 re-samplings. Bayesian analyses were conducted with MRBAYES 3.1 [108] using a GTR + G + I evolutionary model with substitution rates, base frequencies, invariable sites and the shape parameter of the gamma distribution estimated by the program. Two Metropolis-coupled Markov Chain Monte Carlo (*MC*^3^) searches with four chains, three heated and a cold one, were run. The analyses started with a random tree and was run for 5,000,000 generations. Trees and parameters were sampled every 100th generation. The trees were checked to show a standard deviation of split frequencies below 0.05. The first 3,000,000 generations were excluded as the burn-in.

Additionally phylogenetic analyses were conducted with Bayesian inference, using each of the 22 eubacterial species separately with the sampled cyanobacterial subset (58 taxa). Alignments were built using Clustal-X software with default settings [107] and corrected manually. For each phylogenetic analysis two (*MC*^3^) searches were run for 10,000,000 generations using MRBAYES 3.1 [108]. Trees and parameters were sampled every 100th generation. The first 3,000,000 generations being excluded as a burn-in, assuring that the standard deviation of split frequencies were below 0.05 and log-likelihoods of the trees had reached stationarity. Results were compared and *Beggiatoa sp*. was chosen as an outgroup for further analyses.

#### Phylogenetic analyses of a cyanobacterial subset

Sequence alignments of the 16S rRNA gene sequences from the cyanobacterial subset and *Beggiatoa sp*. (59 taxa, 1166 characters) were carried out using Clustal-X with default settings [107] and corrected manually. Whether the cyanobacterial alignment (excluding the outgroup) was substitutionally saturated was tested using the program DAMBE [109,110]. The information-entropy based index of substitutional saturation [111] was used to analyze our alignment of 16S rRNA gene sequences. The test performs only on a maximum of 32 species. Therefore we sampled from our phylogeny 32 representative sequences that span the whole tree, and performed the test introduced by Xia et al. [111](Table 1 and Additional File 4).

Phylogenetic reconstruction was carried out using Bayesian analysis and maximum likelihood. Maximum likelihood analysis was performed using GARLI 0.96 [112] and Bayesian analysis was conducted with MRBAYES 3.1 [108]. The evolutionary model of nucleotide substitution that best fitted the data was obtained by using the Akaike Information Criterion as implemented in Modeltest 3.5 [113]. The selected model was GTR + G + I. Substitution rates, base frequencies, invariable sites and the shape parameter of the gamma distribution were estimated by the program. Fifty maximum likelihood searches were performed. Bootstrap values were calculated from 500 re-samplings of the data set. The bootstrap values were plotted on the best ML-tree using the program SumTrees [114] (Additional File 3).

Bayesian analysis was conducted running two (*MC*^3^) searches, each with four chains, one cold and three heated. Starting with a random tree, analyses were run for 16,616,000 generations each, with trees being sampled every 100th generation. The trees were checked for convergence of parameters (standard deviation of split frequencies below 0.01, effective sample sizes above 200, potential scale reduction factor equal to 1.0) using Tracer v1.4.1 [115] and the program AWTY [116]. Burn-in was set to 3,323,200 generations each, corresponding to the first 20% of the analyses. The average standard deviation of split frequencies was below 0.01 for the remaining 132,929 trees of each run, indicating that steady state of the log-likelihoods was reached.

### Ancestral character state reconstruction

Character state reconstructions were performed using maximum parsimony (MP; Additional File 5) and maximum likelihood criteria as implemented in Mesquite 2.71 [60]. 5,000 trees from each *MC*^3 ^run were randomly chosen from the post burn-in Bayesian sample and combined. Discrete characters were coded into multicellular or unicellular states. The results over 10,000 Bayesian trees were summarized and displayed on the consensus tree of the Bayesian analysis. For maximum likelihood estimates, both the "Markov k-state 1 parameter model" (MK1 model) and "Asymmetrical Markov k-state 2 parameter model" (AsymmMK model) were applied. Rate of change is the only parameter in the MK1 model. The AsymmMK model exhibits two parameters, describing the forward and backward transitions between states. Phylogenetic conservativeness of multicellularity was tested by comparing the observed distribution of parsimony steps across 10,000 randomly chosen trees from the Bayesian analysis against the distribution from 1,000 trees modified from the Bayesian consensus by randomly shuffling the terminal taxa, while keeping the relative proportion of states unaltered. The root was assumed to be at equilibrium. Transition rates for the MK1 and AsymmMK model were estimated by the program. Rates for the latter models presented in Table 2 were estimated for the consensus tree. To explore properties of the data set, character states were additionally reconstructed with manually fixed transition rates (F1-F6; Table 2). The state of the outgroup was excluded from the analyses to avoid biased inferences within the ingroup.

The character states of nodes 3, 4 and 5 of the Bayesian consensus tree were additionally estimated using a reversible jump MCMC search as implemented in BayesTraits [117]. MCMC was run for 30 million iterations, and a burnin set to 50,000. The analysis was run several times with parameters of the evolutionary model being chosen from different prior distributions. In order to determine which model offered the best fitting priors, models were tested using Bayes Factors. A hyperprior approach with mean-values of the exponential priors derived from a uniform distribution between 0 to 10 was determined to fit best the data. The results of the analysis were visualized in Tracer v1.5 [115].

## Authors' contributions

BES and HCB conceived the study; BES gathered data and conducted analyses; BES, HCB, AA designed research and wrote the paper. All authors read and approved the final manuscript.

## Supplementary Material

Additional file 1 **Rooted Bayesian consensus tree of 27 eubacterial species including five cyanobacterial species**. Bayesian analysis of 16S rRNA gene sequences from 27 Eubacteria, based on GTR+I+G substitution model with an archaean outgroup. Posterior probabilities (black) and bootstrap values (red) from 100 re-samplings are displayed at the nodes. Cyanobacteria (blue-green box) are strongly supported as a monophyletic group with *Gloeobacter violaceus *being closest to other eubacterial species. Click here for file

Additional file 2 **Bayesian consensus trees of cyanobacterial subset and different outgroups - newick format**. 22 Bayesian consensus trees with posterior probabilities of a cyanobacterial subset (58 taxa) and different eubacterial outgroups, displayed in newick format. Trees were run for 10,000,000 generations using a GTR+I+G substitution model with the first 3,000,000 generations being discarded as a burn-in. Click here for file

Additional file 3 **Maximum likelihood tree of cyanobacterial subset**. Maximum likelihood analysis of 16S rDNA sequences from 58 cyanobacteria, based on GTR+G+I substitution model, with *Beggiatoa sp*. as an outgroup. Posterior probabilities (> 0.9) in black and bootstrap values (> 50%) in red are shown at the nodes. Posterior probabilities were calculated from 265,858 trees inferred by Bayesian analysis. Bootstrap values were calculated from 500 re-samplings of the data set. Colors define groups: yellow are single-celled cyanobacteria of section I; orange single-celled from section II; green are multicellular, undifferentiated cyanobacteria from section III; blue are multicellular and differentiated bacteria from section IV; and pink from section V. Sections as described by Castenholz 2001 [9]. AC, B, C, E and E1 denote clades discussed in the text. Click here for file

Additional file 4 **Results from the test of substitutional saturation**. Substitutional saturation of the sequences was tested using DAMBE software. The index of substitutional saturation is smaller than the estimated critical value irrespective of the symmetry of the tree. The sequences are therefore not saturated. Click here for file

Additional file 5 **Ancestral character state reconstruction using maximum parsimony**. Summary of results over 10,000 randomly sampled trees from the Bayesian analysis. Uniquely best states were counted and are shown on the Bayesian consensus tree. Possible states are unicellular (yellow) and multicellular (black). At the nodes, probabilities for each character state are represented with a pie chart. The white part in the pie charts indicates fraction of trees where the node was absent, grey parts describe fraction of trees where both states were equally likely. Nodes where transitions occurred were labelled with an asterisk if they show strong support from the phylogenetic analyses. The maximum parsimony analysis produced a similar result compared to the maximum likelihood analysis. A unicellular ancestry for the most recent common ancestor of all cyanobacteria is supported. Nodes 3, 4 and 5 are most frequently optimized as multicellular. Multicellularity has been estimated for nodes 3 and 4 in 6800 trees and for node 5 in 6900 trees. In contrast, single celled states for these nodes have been reported, for node 3 in 13 out of 10,000 trees and for node 4 and 5 in 14 out of 10,000 trees. Five reversals to unicellularity can be detected and at least one reversal to multicellularity. Click here for file

Additional file 6 **Phylogenetic tree of cyanobacteria - newick format**. Phylogenetic tree of 1,254 cyanobacterial sequences including six chloroplasts and six Eubacteria analyzed using maximum likelihood analysis with a GTR+G+I estimated substitution model, conducted with the software RAxML. Click here for file

Additional file 7 **Taxon names of the phylogenetic tree of cyanobacteria**. Species names used in the phylogenetic analysis conducted with RAxML software. Taxon names are ordered by sub-groups as in Figure 1. Click here for file
