# Supplementary figures and images for: The origin of multicellularity in cyanobacteria
Source: BMC Evol Biol. 2011 Feb 14;11:45. doi: 10.1186/1471-2148-11-45 (PMC3271361; doi:10.1186/1471-2148-11-45)

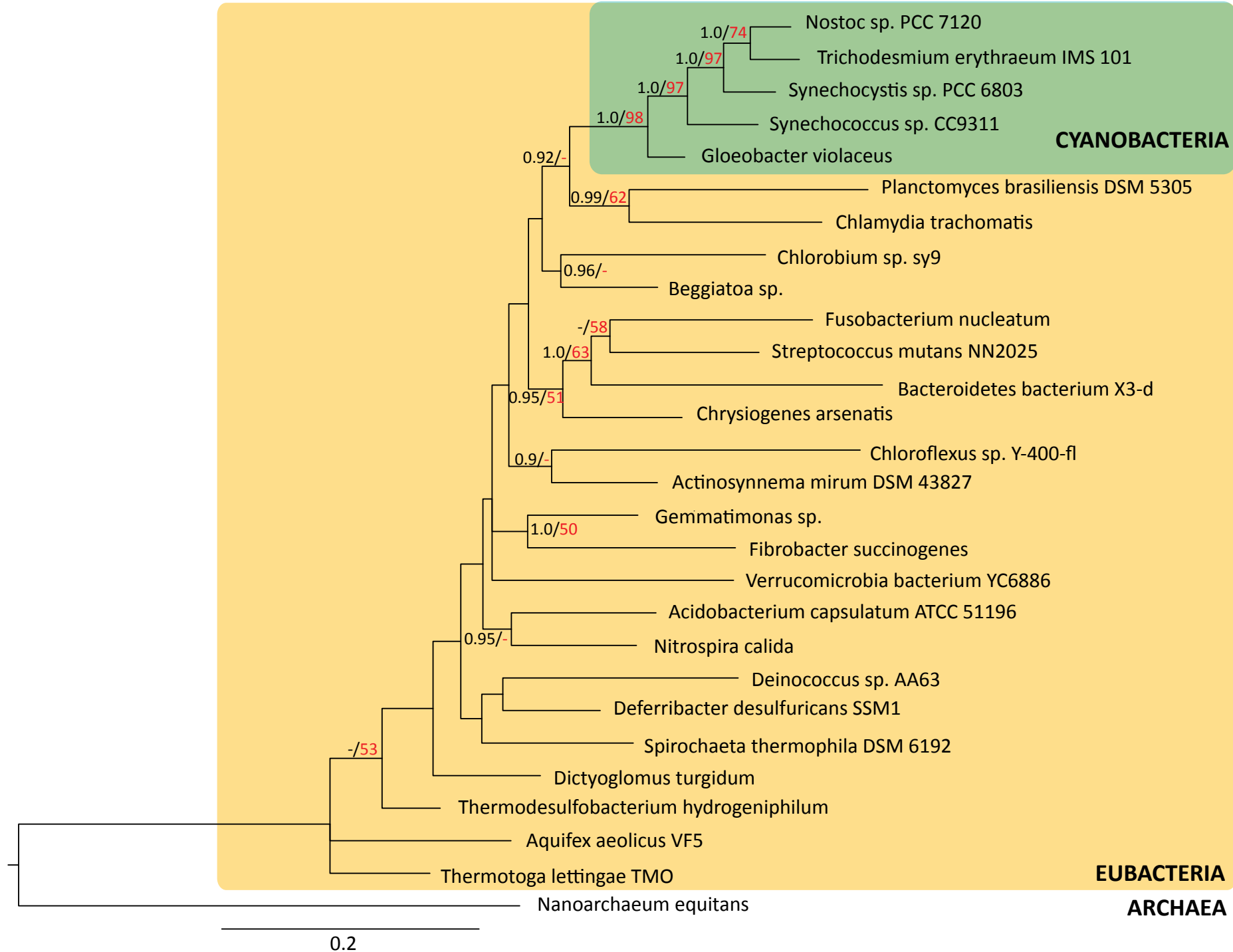

Supplement: Additional file 1 — Rooted Bayesian consensus tree of 27 eubacterial species including five cyanobacterial species. Bayesian analysis of 16S rRNA gene sequences from 27 Eubacteria, based on GTR+I+G substitution model with an archaean outgroup. Posterior probabilities (black) and bootstrap values (red) from 100 re-samplings are displayed at the nodes. Cyanobacteria (blue-green box) are strongly supported as a monophyletic group with Gloeobacter violaceus being closest to other eubacterial species. [file 1471-2148-11-45-S1.pdf]

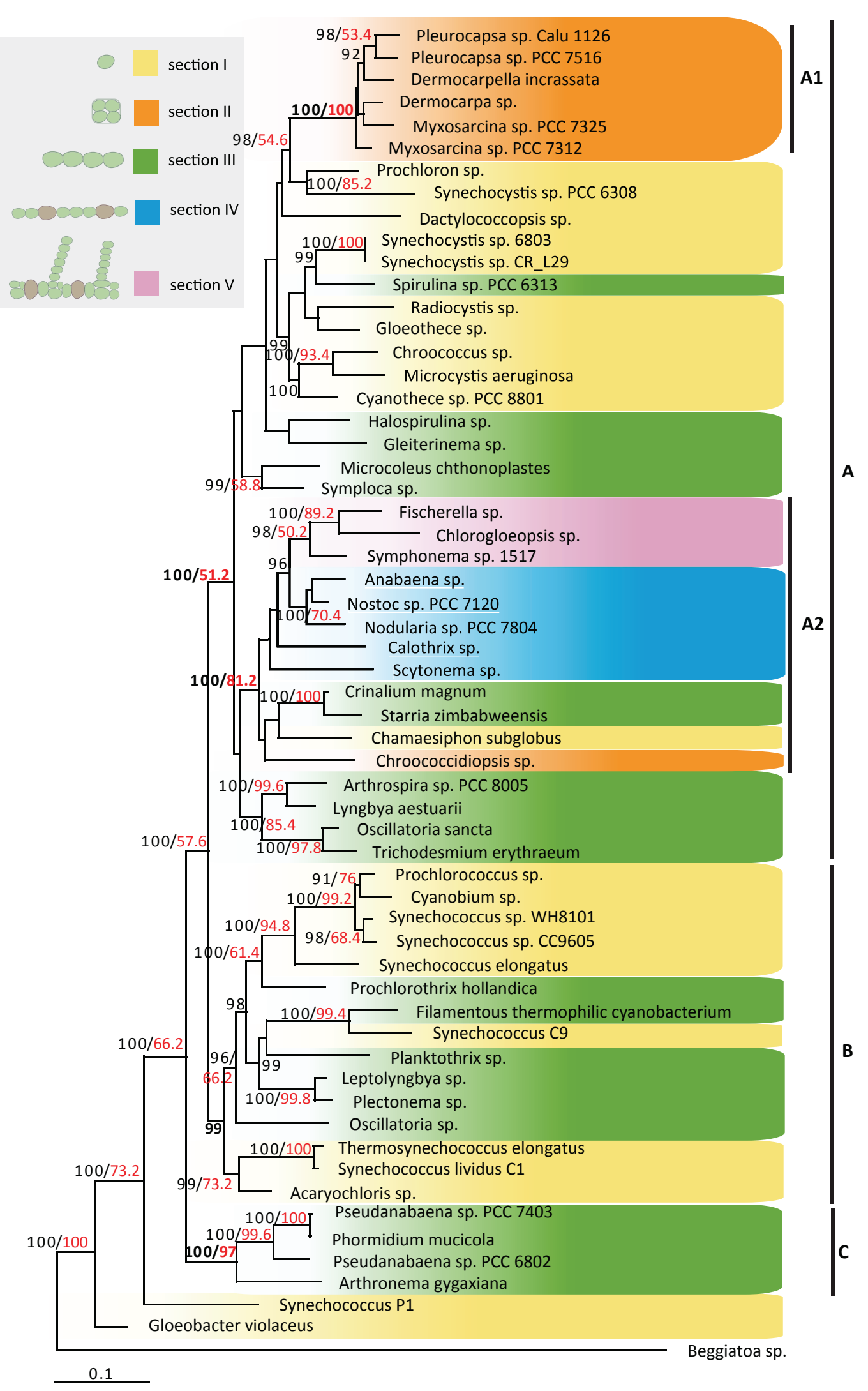

Supplement: Additional file 3 — Maximum likelihood tree of cyanobacterial subset. Maximum likelihood analysis of 16S rDNA sequences from 58 cyanobacteria, based on GTR+G+I substitution model, with Beggiatoa sp. as an outgroup. Posterior probabilities (> 0.9) in black and bootstrap values (> 50%) in red are shown at the nodes. Posterior probabilities were calculated from 265,858 trees inferred by Bayesian analysis. Bootstrap values were calculated from 500 re-samplings of the data set. Colors define groups: yellow are single-celled cyanobacteria of section I; orange single-celled from section II; green are multicellular, undifferentiated cyanobacteria from section III; blue are multicellular and differentiated bacteria from section IV; and pink from section V. Sections as described by Castenholz 2001 [9]. AC, B, C, E and E1 denote clades discussed in the text. [file 1471-2148-11-45-S3.pdf]

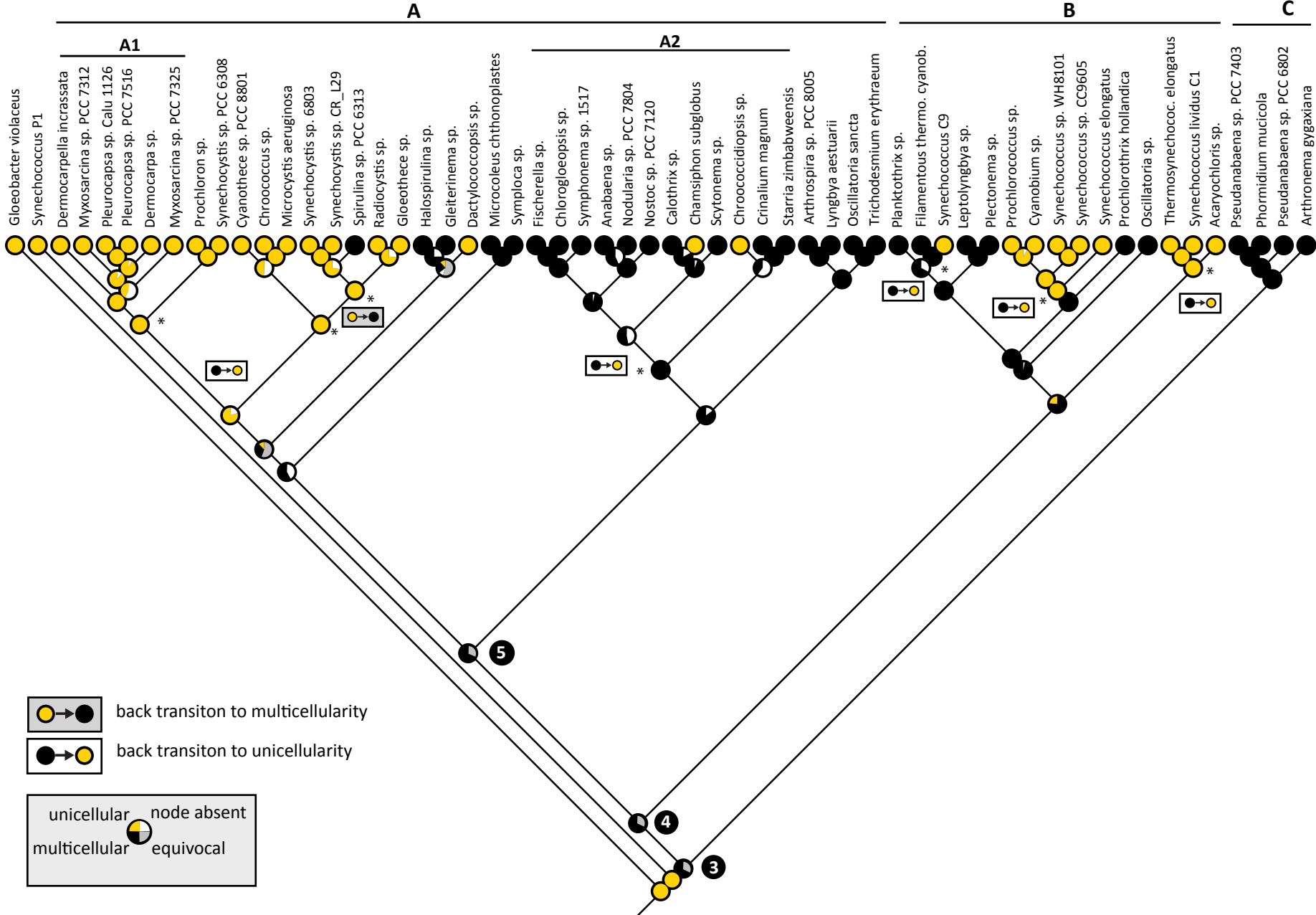

Supplement: Additional file 5 — Ancestral character state reconstruction using maximum parsimony. Summary of results over 10,000 randomly sampled trees from the Bayesian analysis. Uniquely best states were counted and are shown on the Bayesian consensus tree. Possible states are unicellular (yellow) and multicellular (black). At the nodes, probabilities for each character state are represented with a pie chart. The white part in the pie charts indicates fraction of trees where the node was absent, grey parts describe fraction of trees where both states were equally likely. Nodes where transitions occurred were labelled with an asterisk if they show strong support from the phylogenetic analyses. The maximum parsimony analysis produced a similar result compared to the maximum likelihood analysis. A unicellular ancestry for the most recent common ancestor of all cyanobacteria is supported. Nodes 3, 4 and 5 are most frequently optimized as multicellular. Multicellularity has been estimated for nodes 3 and 4 in 6800 trees and for node 5 in 6900 trees. In contrast, single celled states for these nodes have been reported, for node 3 in 13 out of 10,000 trees and for node 4 and 5 in 14 out of 10,000 trees. Five reversals to unicellularity can be detected and at least one reversal to multicellularity. [file 1471-2148-11-45-S5.pdf]
