# Supplementary material for: The origin of multicellularity in cyanobacteria
Source: BMC Evol Biol. 2011 Feb 14;11:45. doi: 10.1186/1471-2148-11-45 (PMC3271361; doi:10.1186/1471-2148-11-45)
Supplement: Additional file 4 — Results from the test of substitutional saturation. Substitutional saturation of the sequences was tested using DAMBE software. The index of substitutional saturation is smaller than the estimated critical value irrespective of the symmetry of the tree. The sequences are therefore not saturated. [file 1471-2148-11-45-S4.pdf]

Additional File 3- Degree of substitutional saturation

Symmetrical tree	
Proportion of invariable sites	0.038
mean H[1]	0.33
Standard error	0.018
ISS[2]	0.173
ISSc[3]	0.756
**Asymmetrical tree**	
ISSc[3]	0.460

[1]Mean entropy for all sites
[2]entropy based index of substitutional saturation
[3]critical value for the index of substitutional saturation
