# Supplementary material for: The origin of multicellularity in cyanobacteria
Source: BMC Evol Biol. 2011 Feb 14;11:45. doi: 10.1186/1471-2148-11-45 (PMC3271361; doi:10.1186/1471-2148-11-45)
Supplement: Additional file 7 — Taxon names of the phylogenetic tree of cyanobacteria. Species names used in the phylogenetic analysis conducted with RAxML software. Taxon names are ordered by sub-groups as in Figure 1. [file 1471-2148-11-45-S7.pdf]

Taxa used to reconstruct a huge phylogenetic tree including 1254 cyanobacterial sequences, six chloroplasts and six eubacterial species

Email:

*Corresponding author

Clade D1 (Anabaena-Nostoc):

Anabaena circinalis AWQC310F 1295 bp
Aphanizomenon flos aquae A8 1295 bp
Anabaena smithii TAC450 1295 bp
Aphanizomenon flos aquae strAph Zayi 1295 bp
Anabaena circinalis 1tu33s12 r strain 1tu33s12 1295 bp
Anabaena ellipsoides Ana HB 1295 bp
Anabaena planctonica TAC424 1295 bp
Anabaena crassa CENA207 1295 bp
Anabaena circinalis AWT205B 1295 bp
Anabaena crassa CENA196 1295 bp
Anabaena circinalis AWQC331C 1295 bp
Anabaena circinalis AWQC332H 1295 bp
Anabaena crassa CENA206 1295 bp
Anabaena flos aquae 04 53 r strain 04 53 1295 bp
Anabaena affinis NIES 40 1295 bp
Anabaena spiroides r strain PMC9702 1295 bp
Anabaena spiroides NIES 79 1295 bp
Anabaena sigmoidea 0tu36s7 r strain 0tu36s7 1295 bp
Anabaena sp1 strAna Ku5 1295 bp
Anabaena planctonica CENA209 1295 bp
Aphanizomenon flos aquae 1tu37s13 r strain 1tu37s13 1295 bp
Aphanizomenon flos aquae AFA 3 1295 bp
Aphanizomenon flos aquae r strain PMC9707 1295 bp
Anabaena sigmoidea 0tu38s4 r strain 0tu38s4 1295 bp
Aphanizomenon flos aquae A7 1295 bp
Aphanizomenon flos aquae 1tu29s19 r strain 1tu29s19 1295 bp
Aphanizomenon flos aquae strAph Ku 1295 bp
Anabaena viguieri TAC433 1295 bp
Aphanizomenon flos aquae 617 1295 bp
Anabaena smithii TAC431 1295 bp
Aphanizomenon flos aquae A1 1295 bp
Aphanizomenon flos aquae AFA 6 1295 bp
Aphanizomenon flos aquae A5 1295 bp
Anabaena solitaria BC Ana 0025 1295 bp
Anabaena flos aquae EH 1 1295 bp

Anabaena flos aquae DC 1 1295 bp
Anabaena spiroides 1tu39s17 r strain 1tu39s17 1295 bp
Anabaena smithii 1tu39s8 r strain 1tu39s8 1295 bp
Anabaena planctonica NIVA CYA 66 1295 bp
Aphanizomenon flos aquae strAph Inba 1295 bp
Anabaena ucrainica TAC449 1295 bp
Anabaena circinalis NIES41 1295 bp
Anabaena spiroides NIES 76 1295 bp
Anabaena circinalis CENA193 1295 bp
Anabaena planctonica TAC421 for 1295 bp
Aphanizomenon flos aquae strAph K2 1295 bp
Anabaena circinalis CENA191 1295 bp
Aphanizomenon flos aquae varKlebahnii r strain 218 1295 bp
Anabaena flos aquae DC 2 1295 bp
Anabaena flos aquae AWQC112D 1295 bp
Anabaena planctonica TAC435 1295 bp
Anabaena circinalis AWQC307C 1295 bp
Aphanizomenon flos aquae r strain PMC9401 1295 bp
Aphanizomenon flos aquae 1tu26s2 r strain 1tu26s2 1295 bp
Anabaena circinalis CENA190 1295 bp
Anabaena planctonica Inba2 1295 bp
Anabaena circinalis AWT001 1295 bp
Aphanizomenon spTR183 r strain TR183 1295 bp
Anabaena spBIR41 1295 bp
Anabaena flos aquae 0tu33s2a r strain 0tu33s2a 1295 bp
Anabaena spBIR259 1295 bp
Aphanizomenon gracile UADFA16 1295 bp
Anabaena spXPORK15F 1295 bp
Aphanizomenon flos aquae strain NIES81 1295 bp
Anabaena spA277 r strain A277 1295 bp
Anabaena sp18B6 1295 bp
Anabaena mendotae 04 11 r strain 04 11 1295 bp
Aphanizomenon gracile strAph NH 5 1295 bp
Anabaena spBIR374 1295 bp
Anabaena spBIR246 1295 bp
Aphanizomenon issatschenkoi strTAC419 1295 bp
Anabaena flos aquae NIVA CYA83/1 r strain CYA83/1 1295 bp
Aphanizomenon gracile strain LMECYA40 1295 bp
Anabaena spBIR202 1295 bp
Anabaena sp1tu34s7 r strain 1tu34s7 1295 bp
Aphanizomenon cfgracile 271 r strain 271 1295 bp
Anabaena lemmermannii strAna Dalai 1295 bp
Anabaena sp318 1295 bp
Anabaena spBIR52 1295 bp
Anabaena spBIR358 1295 bp
Aphanizomenon gracile UADFA11 1295 bp
Anabaena spBIR76 1295 bp
Aphanizomenon flos aquae r strain PMC9706 1295 bp
Anabaena lemmermannii 04 42 r strain 04 42 1295 bp
Aphanizomenon flos aquae 1295 bp
Anabaena oscillarioides BECID22 r strain BECID22 1295 bp
Aphanizomenon issatschenkoi 1313 1295 bp
Anabaena cfcylindrica PMC9705 r strain PMC9705 1295 bp
Anabaena flos aquae 04 40 r strain 04 40 1295 bp
Aphanizomenon issatschenkoi 2312 1295 bp

Anabaena solitaria r strain 82 1295 bp
Anabaena flos aquae 1tu35s12 r strain 1tu35s12 1295 bp
Aphanizomenon spBC Aph 9601 r 1295 bp
Anabaena spBIR348 1295 bp
Anabaena lemmermannii 1tu32s11 r strain 1tu32s11 1295 bp
Anabaena sp315 1295 bp
Anabaena spBIR30 1295 bp
Anabaena flos aquae strain PCC 9302 1295 bp
Anabaena flos aquae 0tu33s15 r strain 0tu33s15 1295 bp
Aphanizomenon issatschenkoi strLEMCYA31 1295 bp
Anabaena sp66A r strain 66A 1295 bp
Anabaena Lemmermannii r strain 202A2 1295 bp
Anabaena lemmermannii BC Ana 0005 1295 bp
Anabaena cffallax CENA208 1295 bp
Anabaena sp90 r strain 90 1295 bp
Aphanizomenon issatschenkoi 473 1295 bp
Anabaena spBIR370B 1295 bp
Anabaena spBIR256 1295 bp
Anabaena lemmermannii TAC437 1295 bp
Anabaena spBIR49 1295 bp
Aphanizomenon gracile 1tu26s16 r strain 1tu26s16 1295 bp
Aphanizomenon gracile 1040 1295 bp
Aphanizomenon flos aquae r strain NIES81 1295 bp
Anabaena sp0tu39s7 r strain 0tu39s7 1295 bp
Anabaena planctonica r strain 71 1295 bp
Anabaena flos aquae 1tu30s4 r strain 1tu30s4 1295 bp
Anabaena spBIR162 1295 bp
Cylindrospermopsis raciborskii for r strain DMKU51018 1295 bp
Cylindrospermopsis raciborskii for r strain DMKU51006 1295 bp
Cylindrospermopsis raciborskii strain Germany 1 1295 bp
Cylindrospermopsis raciborskii BM 1295 bp
Anabaena spXP6B 1295 bp
AF067819 Cylindrospermopsis raciborskii form 2 1295 bp
Cylindrospermopsis raciborskii strain 05E 1295 bp
Cylindrospermopsis raciborskii for r strain CRJ1 1295 bp
Cylindrospermopsis raciborskii strain Brazil 1 1295 bp
Anabaena spBIR361 1295 bp
Anabaena cylindrica XP6B r strain XP6B 1295 bp
Cylindrospermopsis raciborskii strain Germany 2 1295 bp
Cylindrospermopsis raciborskii strain Mk 1295 bp
Anabaena reniformis 07 01 r isolate 07 01 1295 bp
Cylindrospermopsis raciborskii strain LJ 1295 bp
Cylindrospermopsis raciborskii QHSS/NR/CYL/03 1295 bp
Anabaena spBIR5 1295 bp
Cylindrospermopsis raciborskii strain Marau 1 1295 bp
Anabaena spBIR440 1295 bp
Anabaena cylindrica 1295 bp
Anabaena spBECID20 1295 bp
Anabaena spXSP2A 1295 bp
Anabaena spXP34A 1295 bp
Cylindrospermopsis raciborskii strain Florida I 1295 bp
Cylindrospermopsis raciborskii strain Bal 6 1295 bp
Cylindrospermopsis raciborskii strain 23D 1295 bp
Wollea saccata ACCS 045 1295 bp
Cylindrospermopsis raciborskii strain Florida G 1295 bp

Trichormus variabilis HINDAK 2001/4 r strain HINDAK 2001/4 1295 bp
Cylindrospermopsis raciborskii strain Aqc 1295 bp
Raphidiopsis mediterranea HB2 1295 bp
Cylindrospermopsis raciborskii for r strain DMKU51016 1295 bp
Nostoc spAzolla cyanobiont small subunit 1295 bp
Anabaena flos aquae NRC525 17 1295 bp
Cylindrospermopsis raciborskii for r strain DMKU51004 1295 bp
Anabaena spBIR84 1295 bp
Anabaena spBIR272 1295 bp
Anabaena compacta CCAP 1403/24 1295 bp
Cylindrospermopsis raciborskii for r strain DMKU51019 1295 bp
Cylindrospermopsis raciborskii for r strain DMKU51015 1295 bp
Cylindrospermopsis raciborskii for r strain DMKU51009 1295 bp
Cylindrospermopsis raciborskii strain 09A 1295 bp
Cylindrospermopsis raciborskii strain Caia 1295 bp
Anabaena flos aquae NIES 73 1295 bp
AF317629 Anabaena spPCC 7108 1295 bp
Anabaena spXSP36B 1295 bp
Cylindrospermopsis raciborskii for r strain DMKU51017 1295 bp
Anabaena oscillarioides BO HINDAK 1984/43 r strain BO HINDAK 1984/43 1295 bp
Cyanobacterium BECID34 r strain BECID34 1295 bp
Anabaena compacta ANACOM KOR r strain ANACOM KOR 1295 bp
Anabaena cfcylindrica 133 r strain 133 1295 bp
Anabaena spWH School stisolate for r 1295 bp
Anabaena spBIR169 1295 bp
Cylindrospermopsis raciborskii for r strain NIES991 1295 bp
Anabaena spXP15D 1295 bp
Anabaena spBECID23 1295 bp
Anabaena spBIR25 1295 bp
Cylindrospermopsis raciborskii FAS C1 1295 bp
Cylindrospermopsis raciborskii 1295 bp
Cylindrospermopsis raciborskii Goon 1295 bp
Anabaena spXP36D 1295 bp
Anabaena spXP6A 1295 bp
Anabaena spXP6C 1295 bp
Cylindrospermopsis raciborskii for r strain DMKU51003 1295 bp
Anabaena spSSM 00 1295 bp
Cylindrospermopsis raciborskii for r strain NIES993 1295 bp
Cylindrospermopsis raciborskii for r strain DMKU51010 1295 bp
Cylindrospermopsis raciborskii strain Florida F 1295 bp
Anabaena cylindrica DC 3 1295 bp
Anabaena spBIR96 1295 bp
Cylindrospermopsis raciborskii strain 23B 1295 bp
Anabaena sp PCC 7108 1295 bp
Cylindrospermopsis raciborskii strain Florida D 1295 bp
Cylindrospermopsis raciborskii strain Brazil 2 1295 bp
Cylindrospermopsis raciborskii for r strain DMKU51013 1295 bp
Anabaena spXPORK27C 1295 bp
Cylindrospermopsis raciborskii strain 4799 1295 bp
Anabaena spBIR2 1295 bp
Cylindrospermopsis raciborskii strain 24C 1295 bp
Anabaena spKVJF17 1295 bp
Cylindrospermopsis raciborskii for r strain NIES992 1295 bp
Aphanizomenon aphanizomenoides 04 43 r isolate 04 43 1295 bp
Raphidiopsis curvata HB1 1295 bp

Anabaena sedovii ACCS 058 1295 bp
Cylindrospermopsis raciborskii T3 1295 bp
Anabaena aphanizomenoides CENA188 1295 bp
Anabaena flos aquae strain AWQC 264A 1295 bp
Nodularia sphaerocarpa r strain UTEX B 2093 1295 bp
Anabaenopsis spPCC 9215 1295 bp
Nodularia spumigena strain L575 1295 bp
Nodularia spumigena r strain AV1 1295 bp
Nodularia harveyana r strain Bo53 1295 bp
Nodularia sphaerocarpa r strain BECID35 1295 bp
Nodularia spumigena r strain NSPI 05 1295 bp
Nodularia spumigena strain NSOR12 1295 bp
Nodularia harveyana r strain BECID29 1295 bp
Nodularia spumigena strain PCC73104 1295 bp
Nodularia spumigena strain NSKR07 1295 bp
Nodularia sphaerocarpa r strain Up16a 1295 bp
Nodularia sphaerocarpa r strain PCC73104 1295 bp
Nodularia spumigena strain BY1 1295 bp
Calothrix spBECID18 r strain BECID18 1295 bp
Nodularia spumigena r strain HEM 1295 bp
Nodularia spumigena strain HEM 1295 bp
Anabaena cylindrica NIES19 1295 bp
Anabaenopsis abijatae AB2002/18 r strain AB2002/18 1295 bp
Anabaena spBECID19 1295 bp
Anabaena spXP35A 1295 bp
Nodularia spumigena strain UTEX B2092 1295 bp
Nodularia sphaerocarpa 1295 bp
Nodularia spWH Baltic Sea isolate for r 1295 bp
Anabaenopsis cfAB2002/25 abijatae r strain AB2002/25 1295 bp
Nodularia harveyana r strain Lukesova 18/94 1295 bp
Nodularia spumigena strain NSLA01 1295 bp
Nodularia sphaerocarpa PCC 7804 1295 bp
Nodularia spumigena for r 1295 bp
Anabaenopsis elenkinii AB2002/37 r strain AB2002/37 1295 bp
Nodularia harveyana strain PCC7804 1295 bp
Nodularia harveyana strain CDAC1983/300 1295 bp
Nodularia sphaerocarpa r strain BECID36 1295 bp
Anabaenopsis sp1A 1295 bp
Nodularia harveyana strain UTEX B2093 1295 bp
Nodularia spF81 1295 bp
Aphanizomenon spNH 5 1295 bp
Nodularia spumigena strain NSPH02 1295 bp
Nodularia spumigena strain NSBL05 1295 bp
Nodularia spumigena strain NSBR01 1295 bp
Nodularia spPCC 9350 1295 bp
Nodularia spumigena strain NSGL02A10 1295 bp
Cyanospira rippkae 1295 bp
Nodularia spumigena r strain NSOR 12 1295 bp
Nodularia spumigena r strain GR8b 1295 bp
Nodularia spumigena strain NSPH05A14 1295 bp
Anabaenopsis elenkinii AB2006/20 r strain AB2006/20 1295 bp
Anabaena spSKJF11 1295 bp
Anabaenopsis elenkinii AB2002/17 r strain AB2002/17 1295 bp
Nodularia sphaerocarpa r strain Fae19 1295 bp
Nodularia spumigena r strain Huebel 1988/306 1295 bp

Nodularia spumigena r strain F81 1295 bp
Nodularia spumigena r strain AV63 1295 bp
Nodularia spumigena strain NSLA02A4 1295 bp
Anabaena spBECID31 1295 bp
Nodularia spumigena strain HKVV 1295 bp
Nodularia spKAC 17 for r 1295 bp
Nodularia spLEGE06071 1295 bp
Anabaena augstumalis SCMIDKE JAHNKE/4a r strain SCMIDKE JAHNKE/4a 1295 bp
Nodularia spLukesova 1/91 r strain Lukesova 1/91 1295 bp
Anabaena spBECID8 1295 bp
Nodularia harveyana r strain BECID27 1295 bp
Nodularia spumigena GSL023 1295 bp
Anabaena spXPORK36C 1295 bp
Aphanizomenon ovalisporum FAS AP1 1295 bp
AF160256 Anabaena bergii 1295 bp
Umezakia natans TAC101 1295 bp
Nostoc spPeltigera membranacea 4 cyanobiont 1295 bp
Nostoc commune UTEX 584 1295 bp
AF062637 Nostoc spGSV224 1295 bp
Nostoc spLeptogium gelatinosum cyanobiont 1295 bp
Nostoc spUK18 1295 bp
AF027655 Nostoc PCC73102 1295 bp
Nostoc spMollenhauer 1 1 125 1295 bp
Nostoc sp1tu14s8 r strain 1tu14s8 1295 bp
Nostoc commune for r country Japan Ishikawa 1295 bp
Nostoc spDM103 r strain SAG 2028 1295 bp
Nostoc spPeltigera pruinosa cyanobiont 14 1295 bp
Nostoc spLobaria cyanobiont 34 1295 bp
Nostoc spPannaria cfallorhiza cyanobiont NZ 1295 bp
Nostoc spPannaria affleproloma cyanobiont 1a NZ 1295 bp
Nostoc sphaeroides HBHF0604 1295 bp
Nostoc spKVJF4 1295 bp
Nostoc spPeltigera canina 1 cyanobiont 1295 bp
Nostoc spNephroma laevigatum cyanobiont 39 1295 bp
Nostoc spPannaria sphinctrina cyanobiont 1b Ch 1295 bp
Nostoc spPannaria euphylla cyanobiont NZ 1295 bp
Nostoc spNephroma parile cyanobiont 32 1295 bp
Nostoc spLobaria hallii cyanobiont 19 1295 bp
Nostoc spPannaria affleproloma cyanobiont 2 Ch 1295 bp
Nostoc spSAG 29 90 1295 bp
Nostoc spPannaria conoplea cyanobiont No 1295 bp
AF062638 Nostoc ATCC53789 1295 bp
Nostoc commune AHNG0605 1295 bp
Nostoc spMollenhauer 1 1 108 1295 bp
Gloeotrichia echinulata URA3 r environmental colony URA3 1295 bp
Nostoc spSKS2 1295 bp
Nostoc commune for r strain KU002 1295 bp
Nostoc sp9104 r strain 9104 1295 bp
Nostoc punctiforme SAG 71 79 1295 bp
Nostoc spPannaria rubiginella cyanobiont Chile 1295 bp
Nostoc spPeltigera canina 2 cyanobiont 1295 bp
Nostoc spSAG 39 87 1295 bp
Nostoc spMuscicolous cyanobiont 21 1295 bp
Nostoc spPeltigera lepidophora cyanobiont 1295 bp
Nostoc flagelliforme IMGA0408 1295 bp

Nostoc muscorum SAG 57 79 1295 bp
Nostoc spMollenhauer 1 1 115 1295 bp
Nostoc sp8964 3 r strain 8964 3 1295 bp
Nostoc spPannaria mosenii cyanobiont Mex 1295 bp
Nostoc spSKS5 1295 bp
Nostoc punctiforme SAG 69 79 isolation source lichen specimen voucher SAG 69 79 1295 bp
Nostoc spNephroma parile cyanobiont 27 1295 bp
Nostoc spSKJ4 1295 bp
Nostoc entophytum IAM M 267 for 1295 bp
Nostoc spPeltigera didactyla 3 cyanobiont 1295 bp
Nostoc spLobaria pulmonaria cyanobiont 36 1295 bp
Nostoc spPannaria andina 2 cyanobiont Chile 1295 bp
Nostoc spMollenhauer 94 1 1295 bp
Nostoc spPannaria affsphinctrina cyanobiont 1a NC 1295 bp
Nostoc spSKJ1 1295 bp
Nostoc spPannaria araneosa cyanobiont NZ 1295 bp
Nostoc spPannaria fulvescens cyanobiont NZ 1295 bp
Nostoc spKVJF15 1295 bp
Nostoc spPannaria patagonica cyanobiont Ch 1295 bp
Nostoc spNephroma helveticum cyanobiont 1295 bp
Nostoc commune for r country Japan Yamaguchi 1295 bp
Nostoc sp8963 small subunit 1295 bp
Nostoc spSKSF2 1295 bp
Nostoc spPannaria athroophylla cyanobiont Ch 1295 bp
Nostoc spKVSF4 1295 bp
Nostoc commune for r country Japan Okinawa Ryukyu University 1295 bp
Nostoc sp0GU36S01 r strain 0GU36S01 1295 bp
Nostoc calcicola VI r strain VI 1295 bp
Nostoc spPeltigera rufescens 3 cyanobiont 1295 bp
AF317630 Nostoc spAWT 203 1295 bp
Nostoc spKVS1 1295 bp
Nostoc spSKJF2 1295 bp
Nostoc spMollenhauer 1 1 066 1295 bp
Nostoc spPeltigera membranacea 5 cyanobiont 1295 bp
Nostoc spMollenhauer 1 1 106b2 1295 bp
Nostoc spPeltigera rufescens 2 cyanobiont 1295 bp
Nostoc spSticta beauvoisii cyanobiont 1295 bp
Nostoc cfcommune KG 54 for r 1295 bp
Nostoc spNephroma helveticum cyanobiont 37 1295 bp
Nostoc commune for r country Japan Hokkaido 1295 bp
Nostoc commune for r country Japan Saga 1295 bp
Nostoc sp195 A21 1295 bp
Nostoc spKVJF1 1295 bp
Nostoc spPannaria affleproloma cyanobiont 1b NZ 1295 bp
Nostoc spPeltigera canina 4 cyanobiont 1295 bp
Nostoc commune for r 1295 bp
Nostoc spPannaria andina 1 cyanobiont Chile 1295 bp
Nostoc spSKSF3 1295 bp
Nostoc spPeltigera degeni cyanobiont 1295 bp
Nostoc spPCC 9229 small subunit 1295 bp
Nostoc spKVJ20 1295 bp
Nostoc spKVJ2 1295 bp
Nostoc spSKS9 1295 bp
Nostoc punctiforme SAG 68 79 isolation source lichen specimen voucher SAG 68 79 1295 bp
Nostoc spPannaria obscura cyanobiont Aus 1295 bp

Nostoc spPannaria isabellina cyanobiont 2 Ch 1295 bp
Nostoc spSKSL2 1295 bp
Nostoc spPeltigera collina cyanobiont 20 1295 bp
Nostoc spPannaria andina cyanobiont Peru 1295 bp
Gloeotrichia echinulata PYH14 r environmental colony PYH14 1295 bp
Nostoc sp17 1295 bp
Nostoc spNi4 C1 for r 1295 bp
Nostoc spPeltigera membranacea 1 cyanobiont 1295 bp
Nostoc spKVJF8 1295 bp
Nostoc spLukesova 5/96 r strain Lukesova 5/96 1295 bp
Nostoc spLukesova 40/93 r strain Lukesova 40/93 1295 bp
Nostoc spPannaria elixii cyanobiont 2 NZ 1295 bp
Nostoc spMollenhauer 1 1 150b 1295 bp
Nostoc spPeltigera pruinosa cyanobiont 18 1295 bp
Nostoc sp195 A22 1295 bp
Nostoc spCollema crispum cyanobiont 1295 bp
Nostoc spLobaria hallii cyanobiont 1295 bp
Nostoc spNephroma bellum cyanobiont 1295 bp
Nostoc spMassalongia carnosa cyanobiont 1295 bp
Nostoc flagelliforme strSunitezuoqi 1295 bp
Nostoc spAl3 r strain Al3 1295 bp
Nostoc spParmeliella triptophylla cyanobiont 30 1295 bp
Nostoc spPCC 9305 small subunit 1295 bp
Nostoc spSAG 41 87 1295 bp
Nostoc spPannaria durietzii cyanobiont 1 NZ 1295 bp
Nostoc commune 0Brien 02011101 1295 bp
Nostoc spPeltigera rufescens 4 cyanobiont 1295 bp
Nostoc sp8901 1 r strain 8901 1 1295 bp
Nostoc commune for r strain SO 42 1295 bp
Nostoc spSKJ2 1295 bp
Nostoc spPeltigera venosa cyanobiont 15 1295 bp
Nostoc sp8926 r strain 8926 1295 bp
Nostoc spNephroma helveticum cyanobiont 33 1295 bp
Nostoc commune for country Japan Hyogo Akashi 1295 bp
Nostoc spIO 102 I 1295 bp
Nostoc edaphicum X r strain X 1295 bp
Nostoc spSAG 36 92 1295 bp
Nostoc spPeltigera neopolydactyla cyanobiont 1295 bp
Nostoc spKVJF16 1295 bp
Nostoc spSticta fuliginosa cyanobiont 1295 bp
Nostoc commune for r country France Grenoble 1295 bp
Nostoc spHKAR 2 1295 bp
Nostoc spOs1 C1 for r 1295 bp
Anabaena flos aquae RPAN52 clone 1 1295 bp
Anabaena spCH1 1295 bp
Nostoc spCc2 r strain Cc2 1295 bp
Nostoc spCENA105 1295 bp
Trichormus azollae Kom BAI/1983 r strain Kom BAI/1983 1295 bp
Nostoc spPCC 9231 small subunit 1295 bp
Nostoc piscinale BF3 1295 bp
Nostoc spMau15 r strain Mau15 1295 bp
Nostoc spPCC 7906 for r 1295 bp
Anabaena iyengarii RPAN70 clone 1 1295 bp
Anabaena azotica r 1295 bp
Nostoc spPCC 7423 1295 bp

Nostoc spTO1S01 r strain TO1S01 1295 bp
Nostoc spKK 01 for 1295 bp
Anabaena spiroides RPAN57 clone 1 1295 bp
Anabaena iyengarii RPAN6 clone 1 1295 bp
Nostoc spDe1 r strain De1 1295 bp
Nostoc spPCC 9426 r strain PCC 9426 1295 bp
Nostoc sp8941 small subunit 1295 bp
Nostoc sp8916 small subunit 1295 bp
Nostoc linckia vararvense IAM M 30 for r 1295 bp
Anabaena flos aquae UTCC 64 1295 bp
Nostoc ellipsosporum V r strain V 1295 bp
Nostoc sp8938 small subunit 1295 bp
Calothrix spMk1 C1 for r 1295 bp
Anabaena variabilis RPAN16 clone 1 1295 bp
Anabaena sp r 1295 bp
Nostoc spCr4 r strain Cr4 1295 bp
Nostoc muscorum CENA61 1295 bp
Anabaena variabilis for r 1295 bp
Nostoc spPCC 7120 1295 bp
Nostoc spTH1S01 r strain TH1S01 1295 bp
Cylindrospermum spA1345 1295 bp
Anabaena torulosa BF1 1295 bp
Nostoc spYK 01 for 1295 bp
Nostoc spPCC 8976 r strain PCC 8976 1295 bp
Nostoc spPCC 6720 1295 bp
Tolypothrix spIAM M 259 for 1295 bp
Cylindrospermum spPCC 7417 r strain PCC 7417 1295 bp
Nostoc calcicola 99 1295 bp
Nostoc spCENA107 1295 bp
Calothrix spPCC 7101 for r 1295 bp
Nostoc spPCC 8112 r strain PCC 8112 1295 bp
Calothrix elenkinii RPC1 1295 bp
Nostoc sp152 r strain 152 1295 bp
Nostoc spMollenhauer 1 1 067 1295 bp
Nostoc spHK 01 for 1295 bp
Pseudanabaena spMBIC10772 for r strain MBIC10772 1295 bp
Tolypothrix spPCC 7504 r strain PCC 7504 1295 bp
Nostoc spMollenhauer 1 1 088 1295 bp
Nostocaceae cyanobacterium SAG B11 82 1295 bp
Nostoc elgonense TH3S05 r strain TH3S05 1295 bp
Nostoc muscorum CENA18 1295 bp
Tolypothrix spTOL328 r strain TOL328 1295 bp
Tolypothrix spPCC 7415 r strain PCC 7415 1295 bp
Nostoc spKU001 for r 1295 bp
Nostoc spAl1 r strain Al1 1295 bp
Nostoc spCam2S01 r strain Cam2S01 1295 bp
Nostoc calcicola r strain TH2S22 1295 bp
Trichormus doliolum strdoliolum 1 r strain doliolum 1 1295 bp
Nostoc spSKS8 1295 bp
Nostoc verrucosum KU005 for r 1295 bp
Cylindrospermum spCENA33 1295 bp
Nostoc spCENA88 1295 bp
Nostoc carneum BF2 1295 bp
Nostoc carneum IAM M 35 for r 1295 bp
Cylindrospermum licheniforme UTEX 2014 for r 1295 bp

Clade D2 (Calothrix-Fischerella):

Calothrix spBECID30 r strain BECID30 1295 bp
Symphyonema sp1517 r strain 1517 1295 bp
Scytonema spU 3 3 1295 bp
Calothrix D253 r 1295 bp
Rivularia spXSP25A r strain XSP25A 1295 bp
Calothrix spBECID9 r strain BECID9 1295 bp
Rivularia spBECID10 r strain BECID10 1295 bp
Calothrix spBECID1 r strain BECID1 1295 bp
Calothrix spPCC 8909 r strain PCC 8909 1295 bp
Calothrix spBECID26 r strain BECID26 1295 bp
Nostochopsis lobatus 92 1 r strain 92 1 1295 bp
Rivularia spIAM M 261 for r 1295 bp
Calothrix spBIR LS5 r strain BIR LS5 1295 bp
Rivularia atra BIR MGR1 r environmental colony BIR MGR1 1295 bp
Fischerella spCENA161 1295 bp
Rivularia spXP16B r strain XP16B 1295 bp
Stigonema ocellatum SAG 48 90 r strain SAG 48 90 1295 bp
Brasilonema bromeliae SPC 951 1295 bp
Rivularia atra BIR KRIV1 r environmental colony BIR KRIV1 1295 bp
Fischerella thermalis PCC 7521 for r 1295 bp
Hapalosiphon spIAM M 264 for 1295 bp
Westiellopsis prolifica SAG 16 93 r strain SAG 16 93 1295 bp
Rivularia spXP3A r strain XP3A 1295 bp
Chlorogloeopsis spGreenland 5 1295 bp
Symphyonema sp1269 1 r strain 1269 1 1295 bp
Mastigocladopsis repens MORA r strain MORA 1295 bp
Chlorogloeopsis fritschii PCC 6912 for 1295 bp
Chroococcidiopsis spCC4 1295 bp
CfCalothrix spMuscicolous cyanobiont 5 1295 bp
Calothrix spBECID14 r strain BECID14 1295 bp
Brasilonema roberti lammi strlos manantiales1 1295 bp
Calothrix spPCC 7507 r strain PCC 7507 1295 bp
Calothrix spUKK3412 r strain UKK3412 1295 bp
Brasilonema octagenarum UFV OR1 1295 bp
Hapalosiphon hibernicus BZ 3 1 1295 bp
Fischerella muscicola for r 1295 bp
Scytonema spIAM M 262 for 1295 bp
Calothrix desertica PCC 7102 1295 bp
Calothrix spPCC 7715 r strain PCC 7715 1295 bp
Fischerella spCENA19 1295 bp
Scytonema hofmanni PCC 7110 for r 1295 bp
Fischerella PCC7414Calothrix spAHLA9 r strain AHLA9 1295 bp
Fischerella spMV9 1295 bp
Rivularia spBECID12 r strain BECID12 1295 bp
Tolypothrix spCCMP1185 for r 1295 bp
Fischerella spIAM M 263 for 1295 bp
Calothrix spXP11C r strain XP11C 1295 bp
Calothrix spBECID33 r strain BECID33 1295 bp
Calothrix spXP2B r strain XP2B 1295 bp
Calothrix spBECID16 r strain BECID16 1295 bp
Hapalosiphon sp804 1 r strain 804 1 1295 bp
Calothrix spCCMEE 5093 1295 bp
Calothrix spTJ12 UAM 372 1295 bp
Rivularia spXP27A r strain XP27A 1295 bp

Chloroglogloeopsis sp PCC7518Chlorogloeopsis spPCC 9212 for r 1295 bp
Symphyonemopsis spVAPOR1 r strain VAPOR1 1295 bp
Calothrix spBECID6 r strain BECID6 1295 bp
Filamentous thermophilic cyanobacterium tBTRCCn 101 1295 bp
Hapalosiphon welwitschii 1295 bp
Calothrix spMU27 UAM 315 1295 bp
Calothrix spXSP10A r strain XSP10A 1295 bp
Scytonema spHKAR 3 1295 bp
Calothrix spCAL3363 r strain CAL3363 1295 bp
Fischerella spHKAR 5 1295 bp
Chlorogloeopsis fritschii PCC 6912 for r 1295 bp
Mastigocladus laminosus Greenland 8 isolate 8 1295 bp
Rivularia spMU24 UAM 305 1295 bp
Nostochopsis sp89 45 r strain 89 45 1295 bp
Brasilonema spCENA114 1295 bp
Calothrix spPCC 7103 r strain PCC 7103 1295 bp
Calothrix spCAL3361 r strain CAL3361 1295 bp
Calothrix spBECID21 r strain BECID21 1295 bp
Filamentous thermophilic cyanobacterium tBTRCCn 403 1295 bp
Calothrix desertica PCC 7102 for r 1295 bp
Hapalosiphon delicatulus IAM M 266 for 1295 bp
Calothrix spXP9A r strain XP9A 1295 bp
Rivularia spPCC 7116 r strain PCC 7116 1295 bp
Fischerella major NIES 592 for 1295 bp
Stigonematales cyanobacterium AEL04 Oct 6 03 1295 bp
Calothrix spPCC 7714 r strain PCC7714 1295 bp
Brasilonema terrestre CENA116 1295 bp

Clade B1 (Chrooccidiopsis):

Unicellular thermophilic cyanobacterium tBTRCCn 23 1295 bp
Chroococcidiopsis thermalis for r 1295 bp
Chroococcidiopsis spMMG 6 1295 bp
Chroococcidiopsis spBB79 2 r SAG 2023 1295 bp
Chroococcidiopsis spCC3 complete 1295 bp
Chroococcidiopsis spBB84 1 r strain SAG 2025 1295 bp
Chroococcidiopsis spCC2 complete 1295 bp
Chroococcidiopsis spCC1 complete 1295 bp
Unicellular thermophilic cyanobacterium tBTRCCn 28 1295 bp
Chroococcidiopsis spCk4 1295 bp
Chroococcidiopsis spMMG 5 1295 bp
Chroococcidiopsis cubana r strain SAG 39 79 1295 bp
Chroococcidiopsis spBB82 3 r strain SAG 2024 1295 bp
Chroococcidiopsis spBB96 1 r strain SAG 2026 1295 bp

1 single species:

Phormidium autumnale UTEX 1580 1295 bp
Phormidium tergestinum CCALA 155 1295 bp
Lyngbya wollei strCarmichael/Alabama 1295 bp
Phormidium uncinatum SAG 81 79 1295 bp

Clade C1 (Arthrospira-Lyngbya):

Oscillatoria spCYA127 r strain CYA127 1295 bp
Lyngbya polychroa LP5 1295 bp
Microcoleus chthonoplastes CCY9607 1295 bp
Lyngbya majuscula HECT 1295 bp
Lyngbya spVP417a 1295 bp
Trichocoleus sociatus SAG 26 92 1295 bp
Lyngbya polychroa PNG6 68 rrnB 1295 bp
Planktothrix pseudagardhii T19 6 6 for 1295 bp
Lyngbya bouillonii PNG6 41 1295 bp
Microcoleus spDAI 1295 bp
Phormidium murrayi Ant Ph58 1295 bp
Planktothrix agardhii NIVA CYA 68 1295 bp
Microcoleus chtoenoplastes PCC7420
Microcoleus glaciei UTCC 475 1295 bp
Lyngbya sordida NAC8 51 1295 bp
Schizothrix spPNG5 22 1295 bp
Lyngbya polychroa PNG6 51 1295 bp
Planktothrix agardhii NIVA CYA 10 for 1295 bp
Planktothrix pseudagardhii HAB639 1295 bp
Symploca spHBC5 1295 bp
Planktothrix pseudagardhii T1 8 4 for 1295 bp
Symploca atlantica PCC 8002 for r 1295 bp
Microcoleus chthonoplastes EBD 1295 bp
Planktothrix rubescens r strain BC Pla 9303 1295 bp
Planktothrix mougeotii HAB3343 1295 bp
Planktothrix agardhii NIES 595 for 1295 bp
Phormidiaceae cyanobacterium CPER KK1 1295 bp
Oscillatoria spPCC 9018 1295 bp
Planktothrix rubescens PCC 10106 1295 bp
Lyngbya spVP417b 1295 bp
Microcoleus chthonoplastes CCY0002 1295 bp
Planktothrix rubescens CCAP 1459/14 1295 bp
Planktothrix pseudagardhii NIVA CYA 153 for 1295 bp
Oscillatoria spPCC 8926 1295 bp
Pseudanabaenaceae cyanobacterium ANP1 KK1 1295 bp
Lyngbya bouillonii PNG7 29 3 rrnB 1295 bp
Planktothrix pseudagardhii HAB1346 1295 bp
Lyngbya sordida NAC8 49 r 1295 bp
Planktothrix mougeotii TK4 5 for 1295 bp
Planktothrix agardhii NIVA CYA 34 for 1295 bp
Planktothrix sp2 r strain 2 1295 bp
Symploca spCCY0030 1295 bp
Planktothrix rubescens NIVA CYA 151 for 1295 bp
Lyngbya bouillonii PNG5 198 r 1295 bp
Planktothrix spPCC 9214 1295 bp
Planktothrix agardhii CCAP 1459/36 for 1295 bp
Lyngbya majuscula 3L rrnB 1295 bp
Planktothrix agardhii PCC 9637 1295 bp
Lyngbya polychroa PNG6 9 r 1295 bp
Planktothrix pseudagardhii HAB662 1295 bp
Lyngbya sordida NAC8 52 1295 bp
Lyngbya majuscula JHB 1295 bp
Planktothrix agardhii NIVA CYA 30 for 1295 bp
Microcoleus chthonoplastes CCY9606 1295 bp

Planktothrix agardhii PCC 10110 1295 bp
Planktothrix agardhii HAB326 1295 bp
Lyngbya bouillonii PNG7 63 1295 bp
Planktothrix agardhii HAB237 1295 bp
Planktothrix mougeotii TK5 1 for 1295 bp
Planktothrix pseudagardhii HAB1379 1295 bp
Lyngbya polychroa PNG6 45 1295 bp
Lyngbya polychroa PNG6 48 1295 bp
Symploca spVP642c 1295 bp
Symploca PCC 8002 1295 bp
Microcoleus chthonoplastes CCY9608 1295 bp
Planktothrix agardhii NIVA CYA 56/3 for 1295 bp
Planktothrix pseudagardhii r isolate VR1 1295 bp
Microcoleus chthonoplastes MAF 1295 bp
Lyngbya bouillonii PAL08 16 rrnB 1295 bp
Microcoleus chthonoplastes WW11 1295 bp
Microcoleus chthonoplastes LZW 1295 bp
Lyngbya polychroa PNG5 194 rrnB 1295 bp
Oscillatoria spPCC 8954 1295 bp
Lyngbya majuscula 3L 1295 bp
Lyngbya majuscula PAB 1295 bp
Lyngbya majuscula PNG6 221 rrnB 1295 bp
Planktothrix mougeotii TR2 4 for 1295 bp
Lyngbya polychroa PNG6 68 r 1295 bp
Lyngbya polychroa PNG6 38 rrnB 1295 bp
Lyngbya bouillonii PNG7 22 r 1295 bp
Lyngbya bouillonii PNG7 34 1295 bp
Lyngbya majuscula PNG6 221 r 1295 bp
Lyngbya bouillonii PNG7 22 rrnB 1295 bp
Planktothrix agardhii CCAP 1459/23 for 1295 bp
Planktothrix pseudagardhii HAB366 1295 bp
Lyngbya majuscula NAC8 47 1295 bp
Oscillatoria spPCC 8927 1295 bp
Planktothrix spUVFP1 r strain UVFP1 1295 bp
Microcoleus chthonoplastes SAH 1295 bp
Planktothrix sp1LT27S08 r 1295 bp
Lyngbya bouillonii PNG5 198 rrnB 1295 bp
Lyngbya bouillonii PAL08 16 1295 bp
Planktothrix agardhii HAB325 1295 bp
Microcoleus chthonoplastes CCY9605 1295 bp
Microcoleus spHTT U KK5 1295 bp
Microcoleus spSAG 2212 1295 bp
Planktothrix mougeotii HAB002 1295 bp
Lyngbya polychroa PNG6 9 rrnB 1295 bp
Planktothrix pseudagardhii HAB526 1295 bp
Microcoleus chthonoplastes WW6 1295 bp
Oscillatoria spPCC 9631 1295 bp
Microcoleus chthonoplastes CCY9602 1295 bp
Lyngbya sordida NAC8 53 1295 bp
Symploca spVP642b 1295 bp
Symploca spVP642a 1295 bp
Microcoleus sp for r 1295 bp
Lyngbya bouillonii PNG7 29 3 r 1295 bp
Planktothrix agardhii 213 1295 bp
Lyngbya bouillonii PNG7 14 1295 bp

Lyngbya bouillonii PNG7 4 1295 bp
Lyngbya polychroa PNG5 192 1295 bp
Lyngbya sordida NAC8 49 rrnB 1295 bp
Planktothrix rubescens NIES 1266 1295 bp
Microcoleus chthonoplastes CCY9603 1295 bp
Microcoleus chthonoplastes WW3 1295 bp
Symploca spVP377 1295 bp
Planktothrix pseudagardhii HAB1131 1295 bp
Planktothrix pseudagardhii HAB414 1295 bp
Lyngbya polychroa PNG6 2 1295 bp
Planktothrix mougeotii HAB626 1295 bp
Lyngbya polychroa PNG6 57 1295 bp
Symploca atlantica CCY9617 1295 bp
Planktothrix agardhii NIVA CYA 313 for 1295 bp

Clade C2 (Trichodesmium-Oscillatoria):

Geitlerinema spCCY0102 1295 bp
Arthrospira platensis CG590 1295 bp
Oscillatoria miniata NAC8 50 1295 bp
Arthrospira platensis strain UTEX 2340 1295 bp
Oscillatoria spongeliae SI04 40 1295 bp
Hydrocoleum lyngbyaceum HBC7 1295 bp
Phormidium spETS 05 r isolate ETS 05 1295 bp
Oscillatoria prolifera for r 1295 bp
Arthrospira platensis strain NIES 39 1295 bp
Lyngbya hieronymusii varhieronymusii CN4 3 for 1295 bp
Lyngbya cfconfervoides VP0401 1295 bp
Oscillatoria spongeliae SI04 46 1295 bp
Planktothricoides raciborskii INBaOR for 1295 bp
Pseudoscillatoria coralii BgP10 4S 1295 bp
Geitlerinema spCCY9412 1295 bp
Oscillatoria spongeliae SI04 45 1295 bp
Phormidium autumnale Arct Ph5 1295 bp
Phormidium lumbricale UTCC 476 1295 bp
Blennothrix spPNG05 4 1295 bp
Oscillatoria margaritifera NAC8 54 1295 bp
Oscillatoria sancta for r 1295 bp
Oscillatoria spongeliae KR04 3 1295 bp
Oscillatoria spongeliae 36P3 1295 bp
Arthrospira platensis strain SAG 257 80 1295 bp
Ocfcorallinae r 1295 bp
Trichodesmium thiebautii 1295 bp
Trichodesmium sp 1295 bp
Planktothricoides raciborskii NIES 207 for 1295 bp
Oscillatoria spongeliae 32P1 1295 bp
Lyngbya aestuarii PCC 7419 for r 1295 bp
Planktothricoides raciborskii T1 6 2 for 1295 bp
Lyngbya aestuarii for r 1295 bp
Geitlerinema spBBD 1295 bp
Phormidium animale SAG 1459 6 1295 bp
Arthrospira platensis EB 9602 1295 bp
Trichodesmium erythraeum IMS101 for r 1295 bp
Trichodesmium erythraeum 1295 bp
Microcoleus vaginatus UBI KK2 1295 bp

Planktothricoides raciborskii NSLA3 for 1295 bp
Arthrospira maxima FACHB438 complete 1295 bp
Oscillatoria sancta PCC 7515 1295 bp
Geitlerinema spBBD HS217 1295 bp
Arthrospira maxima BJ 2000 1295 bp
Planktothricoides raciborskii OR1 1 for 1295 bp
Lyngbya polychroa LP16 1295 bp
Oscillatoria lutea for 1295 bp
Microcoleus vaginatus PBP D KK1 1295 bp
Microcoleus vaginatus SEV1 KK3 1295 bp
Microcoleus acremanii UTCC 313 1295 bp
Geitlerinema spBBD HS223 1295 bp
Oscillatoria spongeliae SI04 47 1295 bp
Oscillatoria acuminata for r 1295 bp
Oscillatoria spMMG 3 1295 bp
Microcoleus chthonoplastes CCY0602 1295 bp
Trichodesmium tenue 1295 bp
Planktothricoides raciborskii NSLA4 for 1295 bp
Trichodesmium contortum 1295 bp
Tychonema bourrellyi CCAP 1459/11B for 1295 bp
Arthrospira platensis 8005Geitlerinema spA28DM 1295 bp
Phormidium autumnale SAG 35 90 1295 bp
Microcoleus antarcticus UTCC 474 1295 bp
Arthrospira jenneri EB 9604 1295 bp
Microcoleus rushforthii UTCC 296 1295 bp
Oscillatoria spCk2 1295 bp
Oscillatoria spongeliae KR04 1 1295 bp
Microcoleus vaginatus CSI U KK1 1295 bp
Oscillatoria sp195 A20 1295 bp
Phormidium spUTCC 487 1295 bp
Trichodesmium hildebrandtii 1295 bp
Oscillatoria spNAC8 18 1295 bp
Phormidium spKU003 for r 1295 bp
Arthrospira platensis strain PCC 9223 1295 bp
Phormidium autumnale SAG 78 79 1295 bp
Oscillatoria spongeliae SI04 42 1295 bp
Arthrospira platensis strain PCC 9108 1295 bp
Oscillatoria margaritifera NAC8 55 1295 bp
Oscillatoria spongeliae KR04 4 1295 bp
Oscillatoria spongeliae 310P1 1295 bp
Trichodesmium havanum strF34 5 1295 bp
Phormidium autumnale Ant Ph68 1295 bp
Geitlerinema PCC7105 for r 1295 bp
Oscillatoria spongeliae SI04 41 1295 bp
Geitlerinema spFlo1 1295 bp
Phormidium spHBC9 1295 bp
Microcoleus vaginatus SAG 2211 1295 bp
Phormidium cfterebriformis KR2003/25 1295 bp
Geitlerinema spW 1 1295 bp
Microcoleus vaginatus CJI U2 KK2 1295 bp

Clade A1 (Microcystis):

Microcystis spCHAB727 1295 bp
Microcystis ichthyoblabe for r1 1295 bp

MSU66194 Microcystis spKND9506 1295 bp
Microcystis wesenbergii for r 1295 bp
Chroococcus spJJCM 1295 bp
Microcystis spCHAB731 1295 bp
Microcystis wesenbergii NIES 107 1295 bp
Gloeothece sp PCC 6909/1 1295 bp
Microcystis spCHAB728 1295 bp
Cyanothece sp PCC 8801 1295bp
Microcystis spCHAB729 1295 bp
MAU40331 Microcystis spAWT139 1295 bp
Merismopedia glauca 0BB39S01 r strain 0BB39S01 1295 bp
MWU40334 Microcystis wesenbergii NIES112 r 1295 bp
M glauca strain B1448 1 rDNA 1295 bp
Gmembranacea r 1295 bp
Microcystis wesenbergii DNA for 1295 bp
Microcystis aeruginosa strain 038 1295 bp
Microcystis aeruginosa for strain NIES 604 1295 bp
Microcystis sp269 r strain 269 1295 bp
Microcystis viridis NIES 102 1295 bp
Cyanothece spWH 8904 1295 bp
Microcystis spCHAB1447 1295 bp
Crocosphaera watsonii WH 8501 1295 bp
AF193247 Symbiont of Climacodium frauenfeldianum 1295 bp
Microcystis novacekii for r 1295 bp
Cyanothece spPCC 7424 1295 bp
MAU40338 Microcystis aeruginosa PCC7005 r 1295 bp
Snowella litoralis 1LT47S05 r strain 1LT47S05 1295 bp
Microcystis aeruginosa NPCD 1 1295 bp
Microcystis aeruginosa 0BF29S03 r 1295 bp
Microcystis spCHAB1449 1295 bp
Snowella litoralis 0TU37S04 r strain 0TU37S04 1295 bp
Cyanothece spWH 8902 1295 bp
Microcystis aeruginosa for r isolate TAC86 1295 bp
Pleurocapsa PCC7327 for r 1295 bp
Microcystis spGL260735 1295 bp
Microcystis ichthyoblabe 0BB35S01 r 1295 bp
Microcystis spCHAB657 1295 bp
Microcystis novacekii for r isolate TAC65 1295 bp
Cyanothece sp r strain PCC 7424 1295 bp
MAU03402 Microcystis aeruginosa PCC7806 r 1295 bp
Microcystis aeruginosa 0BB35S02 r 1295 bp
Synechocystis PCC6805 for r 1295 bp
MAU40339 Microcystis aeruginosa PCC7820 r 1295 bp
Microcystis novacekii for r isolate TAC20 1295 bp
Microcystis sp for r isolate 4B3 1295 bp
Microcystis aeruginosa strain SPC 777 1295 bp
Microcystis sp for r isolate T17 1 1295 bp
Microcystis aeruginosa r isolate MK10 10 1295 bp
Microcystis aeruginosa DNA for 1295 bp
Microcystis spCHAB726 1295 bp
Microcystis viridis DNA for 1295 bp
Microcystis sp130 r strain 130 1295 bp
Microcystis aeruginosa for 1295 bp
Synechocystis spCCALA 700 1295 bp
Snowella rosea 1LM40S01 r strain 1LM40S01 1295 bp

Woronichinia naegeliana 0LE35S01 r strain 0LE35S01 1295 bp
Microcystis ichthyoblabe for r 1295 bp
Aphanothece sacrum for r 1295 bp
Synechocystis PCC6803 MAU03403 Microcystis aeruginosa NIES89 r 1295 bp
MVU40332 Microcystis viridis NIES 102 complete 1295 bp
Radiocystis spJJ30 1295 bp
MWU40333 Microcystis wesenbergii NIES 107 complete 1295 bp
MAU40340 Microcystis aeruginosa PCC 7941 complete 1295 bp
Microcystis sp for r isolate T1 4 1295 bp
Microcystis spGL280641 r strain GL280641 1295 bp
Aphanocapsa spHBC6 1295 bp

Clade C3 (Spirulina):

Spirulina spstrain MPI S4 r 1295 bp
Spirulina major 0BB36S18 r strain 0BB36S18 1295 bp
Spirulina subsalsa IAM M 223 for 1295 bp
Spirulina PCC6313
Spirulina spGLS010 1295 bp
Halospirulina sp CCC Baja 95 Cl 3 r 1295 bp
Spirulina spstrain CCC Snake PY 85 r 1295 bp
Halospirulina tapeticola CCC Baja 95 Cl 2 1295 bp
Halospirulina sp MPI S3 r 1295 bp

Clade A2 (Synechocystis):

Synechocystis PCC6308 1295 bp
Synechococcus spHOG 1295 bp
Cyanobacterium spMBIC10216 for r 1295 bp
Synechococcus spPCC 8807 1295 bp
Cyanothece sp115 1295 bp
Synechococcus spPH40 1295 bp
Synechococcus sp for r strain PCC73109 1295 bp
Halothece spPCyano42 1295 bp
Cyanothece sp r strain PCC 7418 1295 bp
Synechococcus elongatus CCMP1630 1295 bp
Synechococcus spT71 1295 bp
Synechococcus spUH7 1295 bp
Euhalothece spZ M001 1295 bp
Dactylococcopsis sp PCC 8305 1295 bp
Euhalothece sp r strain MPI 96N304 1295 bp
Cyanothece sp109 1295 bp
Rubidibacter lacunae KORDI 51 2 1295 bp
Oscillatoria rosea IAM M 220 for 1295 bp
Cyanothece spGSL007 1295 bp
Synechocystis trididemni for r 1295 bp
Euhalothece sp r strain MPI 95AH10 1295 bp
Cyanothece sp113 1295 bp
Phormidium spMBIC10210 for r strain MBIC10210 1295 bp
Halothece spPCC 7418 1295 bp
Prochloron sp for 1295 bp
Cyanothece sp104 1295 bp
Synechococcus sp for r strain PCC7003 1295 bp
Synechococcus sp r strain PCC 7002 1295 bp

Euhalothece sp r strain MPI 95AH13 1295 bp
Synechococcus sp for r strain PCC7117 1295 bp
Halothece sp r strain MPI 96P605 1295 bp

Clade B2 (Pleurocapsa):

Pleurocapsa minor r strain SAG 4 99 1295 bp
Dermocarpella incrassata r strain SAG 29 84 1295 bp
Dermocarpa spMBIC10768 for r 1295 bp
Chroococcidiopsis spPCC 6712 r strain PCC 6712 1295 bp
Myxosarcina PCC 7325 1295 bp
Chroococcidiopsis PCC6712 for r 1295 bp
Stanieria PCC7301 for r 1295 bp
Myxosarcina PCC 7312 1295 bp
Dermocarpa spMBIC10004 for r 1295 bp
Pleurocapsa spCALU 1126 1295 bp
Pleurocapsa spOU 12 1295 bp
Pleurocapsa PCC7319 for r 1295 bp
Pleurocapsa sp r 1295 bp
Pleurocapsa spOU 11 1295 bp
Chroococcidiopsis spCCMP1489 r strain CCMP1489 1295 bp

single species:

Xenococcus spCR 15M 1295 bp
Stanieria cyanosphaera PCC 7437 1295 bp
Stanieria cyanosphaera for r 1295 bp

Clade A3 (Chroococcus):

Chroococcus spJJCV r strain JJCV 1295 bp
Chroococcus spCCALA 702 1295 bp
Chroococcus spCCALA 701 1295 bp
Chroococcus spCCALA 057 1295 bp
Chroococcus cfmembraninus CCALA 054 1295 bp
Gloeocapsa PCC73106 for r 1295 bp
Limnococcus limneticus Svet06 1295 bp
Chroococcus minutus CCALA 055 1295 bp

Clade A4 (Synechococcus):

Cyanobacterium 5X15 r 1295 bp
Synechococcus spKORDI 70 1295 bp
Prochlorococcus marinus subsppastoris stTL2 1295 bp
Synechococcus spKORDI 15 1295 bp
Synechococcus spKORDI 49 1295 bp
Synechococcus sp for r strain PCC7001 1295 bp
AF098372 Synechococcus like strABRAXAS 1295 bp
Cyanobium spJJ2 3 r strain JJ2 3 1295 bp
Cyanobium sp0BB42S04 r strain 0BB42S04 1295 bp
MHU40336 Microcystis holsatica NIES43 r 1295 bp
Cyanobium spJJ9 A3 r strain JJ9 A3 1295 bp
Synechococcus spKORDI 63 1295 bp

Synechococcus PCC7943 1295 bp
Synechococcus spBN39 1295 bp
AF098370 Synechococcus like strACE 1295 bp
Synechococcus spHOS 1295 bp
AF115268 Prochlorococcus spMIT9201 1295 bp
Synechococcus spTAGS 1295 bp
AF001467 Prochlorococcus spNATL2A 1295 bp
Prochlorococcus marinus strTAK9803 2 1295 bp
Syn CC9605Synechococcus spCENA108 1295 bp
Synechococcus spKORDI 18 1295 bp
Syn WH8101Synechococcus spKORDI 11 1295 bp
AF098374 Synechococcus like strP212 1295 bp
Synechococcus spKORDI 12 1295 bp
Cyanobium spJJ27STR r strain JJ27STR 1295 bp
AF001470 Prochlorococcus spNATL2 1295 bp
Synechococcus spRS9921 1295 bp
Synechococcus spKORDI 13 1295 bp
Cyanobium spJJ9 C6 r strain JJ9 C6 1295 bp
Cyanobium spJJ17 5 r strain JJ17 5 1295 bp
AF081834 Synechococcus WH7803 1295 bp
AF001474 Prochlorococcus spTATL1A 1295 bp
Cyanobium spJJ5 5 r strain JJ5 5 1295 bp
Synechococcus spOli31 1295 bp
Synechococcus spMinos11 1295 bp
Synechococcus spPS838 1295 bp
Cyanobium spJJM10A4 r strain JJM10A4 1295 bp
Cyanobium spY0011 1295 bp
Synechococcus sp1tu39s01 r strain 1tu39s01 1295 bp
Synechococcus spBN31 1295 bp
Cyanobium spJJ12A2 r strain JJ12A2 1295 bp
Synechococcus spMBIC10459 for r 1295 bp
Cyanobium spJJ19B5 r strain JJ19B5 1295 bp
Synechococcus spKORDI 54 1295 bp
Synechococcus sp0tu28s07 r strain 0tu28s07 1295 bp
Synechococcus spKORDI 16 1295 bp
Cyanobium spJJ15 13 r strain JJ15 13 1295 bp
AF115270 Prochlorococcus spMIT9211 1295 bp
P marinus for 1295 bp
Synechococcus spEW15 1295 bp
MEU40335 Microcystis elabens NIES42 r 1295 bp
AF001479 Synechococcus WH8103 1295 bp
Synechococcus spBAC 9803 1295 bp
Synechococcus spBAC 9810 1295 bp
Synechococcus spKORDI 71 1295 bp
Cyanobium spJJM10D5 r strain JJM10D5 1295 bp
Synechococcus spKORDI 30 1295 bp
Cyanobium spJJ11D3 r strain JJ11D3 1295 bp
Aphanothece sp0BB21S01 r strain 0BB21S01 1295 bp
Synechococcus spPS723 1295 bp
Synechococcus spMBIC10007 for r 1295 bp
Synechococcus spMBIC10456 for r 1295 bp
Synechococcus spBE0807I 1295 bp
Synechococcus PCC9005 1295 bp
Synechococcus PCC7009 1295 bp
Synechococcus spWH 5701 1295 bp

Synechococcus spRS9918 1295 bp
Synechococcus spMBIC10089 for r 1295 bp
AF098373 Synechococcus like strP211 1295 bp
AF053397 Prochlorococcus spMIT9303 1295 bp
Synechococcus sp0BB26S03 r strain 0BB26S03 1295 bp
AF001478 Synechococcus WH7805 1295 bp
Synechococcus spPS715 1295 bp
Synechococcus sp0BB22S0 r strain 0BB22S05 1295 bp
Synechococcus spC129 1295 bp
Microcystis elabens DNA for 1295 bp
Synechococcus spMLCB 1295 bp
Cyanobium spLB03 1295 bp
Microcystis holsatica DNA for 1295 bp
AF098371 Synechococcus like strPENDANT 1295 bp
Synechococcus spWH 8103 1295 bp
Synechococcus spWH 8018 1295 bp
Synechococcus spKORDI 52 1295 bp
Synechococcus PCC7918 1295 bp
AF001468 Prochlorococcus spMIT9107 1295 bp
AF001472 Prochlorococcus spGP2 1295 bp
Synechococcus spWH 7803 1295 bp
Prochlorococcus marinus strEQPAC1 1295 bp
Prochlorococcus spNATL1 1295 bp
Cyanobium spJJ12 6 r strain JJ12 6 1295 bp
Synechococcus spPS680 1295 bp
Synechococcus spAlmo3 1295 bp
Cyanobium spJJ7 9 r strain JJ7 9 1295 bp
Synechococcus spKORDI 17 1295 bp
Synechococcus spNAN 1295 bp
Cyanobium spNS01 1295 bp
Synechococcus spMBIC10613 for r strain MBIC10613 1295 bp
Cyanobium spJJ22 10 r strain JJ22 10 1295 bp
Synechococcus spRS9920 1295 bp
Synechococcus spKORDI 56 1295 bp
Synechococcus spMinos12 1295 bp
AF001475 Prochlorococcus spTATL1B 1295 bp
Synechococcus spIOCAS0401 1295 bp
Prochlorococcus marinus subsppastoris stTL1 1295 bp
Synechococcus spTAG 1295 bp
Synechococcus spWH 8020 1295 bp
Synechococcus spUBR 1295 bp
Synechococcus spKORDI 36 1295 bp
Synechococcus spKUAC 3043 1295 bp
Cyanobium spPCC 6904 1295 bp
Cyanobium spJJ13 6 r strain JJ13 6 1295 bp
AF115269 Prochlorococcus spMIT9202 1295 bp
AF053396 Prochlorococcus spMIT9302 1295 bp
Synechococcus spACT9807 1295 bp
Prochlorococcus spCCMP1426 1295 bp
Synechococcus spWH 8016 1295 bp
Synechococcus spKORDI 50 1295 bp
Synechococcus spCCMP839 1295 bp
Cyanobium spGSL004 1295 bp
Syn PCC6301 Cyanobium spJJ10 3 r strain JJ10 3 1295 bp
Synechococcus spT7cc1 1295 bp

AF053398 Prochlorococcus spMIT9312 1295 bp
Cyanobium spJJ19 11 r strain JJ19 11 1295 bp
Synechococcus spGL150636 r strain GL150636 1295 bp
Cyanobium spJJNV r strain JJNV 1295 bp
AF053399 Prochlorococcus spMIT9313 1295 bp
AF115271 Prochlorococcus spMIT9215 1295 bp
Synechococcus spPS845 1295 bp
Cyanobium spJJM10D4 r strain JJM10D4 1295 bp
Synechococcus spKORDI 65 1295 bp
Synechococcus spKORDI 78 1295 bp
Synechococcus spKORDI 42 1295 bp
Cyanobium spJJ22K r strain JJ22K 1295 bp
Merismopedia spCENA106 1295 bp
Synechococcus spPS840 1295 bp
Synechococcus spIOCAS0402 1295 bp
Cyanodictyon spJJCD r strain JJCD 1295 bp
AF001471 Prochlorococcus spPAC1 1295 bp
Synechococcus PCC7920 1295 bp
Cyanobium spJJ9 5 r strain JJ9 5 1295 bp
Cyanobium spJJR2A5 r strain JJR2A5 1295 bp
Synechococcus spWH 8012 1295 bp
Synechococcus spPCC 8966 1295 bp
Cyanobium spJJ8 4 r strain JJ8 4 1295 bp

Clade AC1 :

Leptolyngbya sp 1295 bp
Synechococcus spMBIC10598 for r strain MBIC10598 1295 bp
MSU66194 Microcystis spKND9506 1295 bp
Microcystis wesenbergii for r 1295 bp
Leptolyngbya spCENA112 1295 bp
FilthermocyanobacteriumLeptolyngbya tenerrima UTCC 77 1295 bp
Leptolyngbya spCENA103 1295 bp
CfLeptolyngbya spGreenland 9 1295 bp
Limnothrix spB15 1295 bp
Leptolyngbya spFYG 1295 bp
MWU40334 Microcystis wesenbergii NIES112 r 1295 bp
Filamentous thermophilic cyanobacterium tBTRCCn 102 1295 bp
Calothrix spKVSF5 1295 bp
Arthronema africanum SAG 12 89 for 1295 bp
Limnothrix redekei 165c r strain 165c 1295 bp
Leptolyngbya spHBC3 1295 bp
L foveolarum r 1295 bp
AF170757 LPP group cyanobacterium QSSC5cya 1295 bp
Phormidium spIAM M 99 for 1295 bp
Leptolyngbya angustata UTCC 473 1295 bp
Oscillatoriales cyanobacterium OU 4 1295 bp
Leptolyngbya ant lh52 1Leptolyngbya spOU 14 1295 bp
Leptolyngbya spBN34 1295 bp
Phormidium spOU 10 1295 bp
Oscillatoriales cyanobacterium UVFP2 r strain UVFP2 1295 bp
Microcystis wesenbergii DNA for 1295 bp
Limnothrix spCENA110 1295 bp
Leptolyngbya spOU 8 1295 bp
Filamentous cyanobacterium GSL035 1295 bp

Microcystis sp269 r strain 269 1295 bp
Halomicronema spSCyano39 1295 bp
Starria zimbabweensis SAG 74 90 for r 1295 bp
Limnothrix spCENA109 1295 bp
Pseudanabaena tremula UTCC 471 1295 bp
Synechococcus spIR11 1295 bp
Chamaesiphon PCC7430Leptolyngbya spBN44 1295 bp
Phormidium spMBIC10818 for r strain MBIC10818 1295 bp
PlectonemMAU03402 Microcystis aeruginosa PCC7806 r 1295 bp
Leptolyngbya spNi6 C2 for r 1295 bp
CfLeptolyngbya spGreenland 10 1295 bp
Leptolyngbya spGreenland 7 1295 bp
Halomicronema spTFEP1 1295 bp
Leptolyngbya spBN43 1295 bp
Synechococcus C9Pseudanabaenaceae cyanobacterium PBP U KK1 1295 bp
Microcystis aeruginosa DNA for 1295 bp
L boryanum 1295 bp
Oscillatoriales cyanobacterium JSC 1 1295 bp
Filamentous thermophilic cyanobacterium tBTRCCn 408 1295 bp
Synechococcus sp for r strain PCC7335 1295 bp
Filamentous thermophilic cyanobacterium tBTRCCn 302 1295 bp
Leptolyngbya spOU 13 1295 bp
LPP group MBIC10086 for r 1295 bp
Oscillatoria spIAM M 117 for 1295 bp
Phormidium spMBIC10002 for r 1295 bp
Limnothrix redekei 165a r strain 165a 1295 bp
AF170758 LPP group cyanobacterium QSSC8cya 1295 bp
Phormidium spSAG 37 90 1295 bp
Leptolyngbya spMMG 1 1295 bp
Leptolyngbya spCENA104 1295 bp
Planktothrix sp FP1 1295 bp
LPP group MBIC10597 for r 1295 bp
AF170756 LPP group cyanobacterium QSSC3cya 1295 bp
Crinalium magnum SAG 34 87 for 1295 bp
MAU40340 Microcystis aeruginosa PCC 7941 complete 1295 bp
Leptolyngbya spITAC101 1295 bp
Filamentous thermophilic cyanobacterium tBTRCCn 407 1295 bp

Clade A5 (Acaryochloris):

Acaryochloris spAwaji 1 for r 1295 bp
Acaryochloris marina strain MBIC 11017 1295 bp
Synechococcus spPCC 6312 1295 bp
Acaryochloris spJJ7 5 r strain JJ7 5 1295 bp
Syn lividusC1
Thermosynechococcus BP 1
Acaryochloris marina for r strain MBIC11017 1295 bp
Synechococcus PCC6717 1295 bp
Acaryochloris spJJ8A6 r strain JJ8A6 1295 bp

Clade C4 (Pseudanabaena):

Anabaena spiroides NIES 78 1295 bp
Filamentous cyanobacterium 72 1 1295 bp

Pseudanabaena PCC7367 for r 1295 bp
Synechococcus spPCC 7502 1295 bp
Pseudanabaena PCC7403 for r 1295 bp
Arthronema gygaxiana UTCC 393 1295 bp
Pseudanabaena sp63 1 1295 bp
Oscillatoria limnetica strain MR1 r 1295 bp
Pseudanabaena sp1tu24s9 r strain 1tu24s9 1295 bp
Phormidium mucicola IAM M 221 1295 bp
Pseudanabaena PCC6802 1295 bp
Pseudanabaena PCC7408 for r 1295 bp
Limnothrix redekei NIVA CYA 227/1 for 1295 bp
Pseudanabaena spPCC 7403 1295 bp
Pseudanabaena sp0tu30s18 r strain 0tu30s18 1295 bp
Pseudanabaena PCC6903 for r 1295 bp

Chloroplasts:

Oedogonium cardiacum chloroplast
Prochlorales cyanobacterium EV 7 1295 bp
Glaucocystis nostochinearum plastid 16S rRNA gene
Parachlorella kessleri chloroplast
Stigeoclonium helveticum strain UTEX 441 chloroplast
Porphyra yezoensis chloroplast

Single species at the basis:

Gloeobacter violaceus PCC 7421
Synechococcus sp. PCC 7336 1295 bp
Synechococcus sp. P1

Non-cyanobacteria:

Chlorobium sp.
Agrobacterium tumefaciens
Beggiatoa sp. 'Chiprana'
Candidatus Chlorothrix halophila
Thiobacillus prosperus
Escherichia coli HS
